# A comprehensive model for assessing and classifying patients with thrombotic microangiopathy: the TMA-INSIGHT score

**DOI:** 10.1186/s12959-023-00564-6

**Published:** 2023-11-22

**Authors:** Vanessa Vilani Addad, Lilian Monteiro Pereira Palma, Maria Helena Vaisbich, Abner Mácola Pacheco Barbosa, Naila Camila da Rocha, Marilia Mastrocolla de Almeida Cardoso, Juliana Tereza Coneglian de Almeida, Monica AP de Paula de Sordi, Juliana Machado-Rugolo, Lucas Frederico Arantes, Luis Gustavo Modelli de Andrade

**Affiliations:** 1 Department of Internal Medicine - UNESP, Univ Estadual Paulista, Rubião Jr, s/n, Botucatu/SP, 18618-687 Brazil; 2 Department of Pediatrics, Universidade Estadual de Campinas, R. Tessália Vieira de Camargo, 126 - Cidade Universitária, Campinas/SP, 13083-887 Brazil; 3 Pediatric Nephrology Service, Child Institute, University of São Paulo, Av. Dr. Enéas Carvalho de Aguiar, 647, São Paulo, SP 05403-000 Brazil; 4 Health Technology Assessment Center of Hospital das Clínicas - HCFMB, Botucatu, SP, Brazil

**Keywords:** Thrombotic microangiopathy, Thrombotic Thrombocytopenic Purpura, Shiga toxin-mediated hemolytic uremic syndrome, Complement mediated TMA, Atypical hemolytic uremic syndrome

## Abstract

**Background:**

Thrombotic Microangiopathy (TMA) is a syndrome characterized by the presence of anemia, thrombocytopenia and organ damage and has multiple etiologies. The primary aim is to develop an algorithm to classify TMA (TMA-INSIGHT score).

**Methods:**

This was a single-center retrospective cohort study including hospitalized patients with TMA at a single center. We included all consecutive patients diagnosed with TMA between 2012 and 2021. TMA was defined based on the presence of anemia (hemoglobin level < 10 g/dL) and thrombocytopenia (platelet count < 150,000/µL), signs of hemolysis, and organ damage. We classified patients in eight categories: infections; Malignant Hypertension; Transplant; Malignancy; Pregnancy; Thrombotic Thrombocytopenic Purpura (TTP); Shiga toxin-mediated hemolytic uremic syndrome (STEC-SHU) and Complement Mediated TMA (aHUS). We fitted a model to classify patients using clinical characteristics, biochemical exams, and mean arterial pressure at presentation.

**Results:**

We retrospectively retrieved TMA phenotypes using automatic strategies in electronic health records in almost 10 years (n = 2407). Secondary TMA was found in 97.5% of the patients. Primary TMA was found in 2.47% of the patients (TTP and aHUS). The best model was LightGBM with accuracy of 0.979, and multiclass ROC-AUC of 0.966. The predictions had higher accuracy in most TMA classes, although the confidence was lower in aHUS and STEC-HUS cases.

**Conclusion:**

Secondary conditions were the most common etiologies of TMA. We retrieved comorbidities, associated conditions, and mean arterial pressure to fit a model to predict TMA and define TMA phenotypic characteristics. This is the first multiclass model to predict TMA including primary and secondary conditions.

**Supplementary Information:**

The online version contains supplementary material available at 10.1186/s12959-023-00564-6.

## Background

Thrombotic Microangiopathy (TMA) is a syndrome characterized by the presence of anemia and thrombocytopenia as a consequence of microthrombi formation, resulting in organ damage by ischemic injury [1]. TMA represents a clinical manifestation of a histological condition marked by several features, including arteriolar and capillary thickening, detachment and swelling of endothelial cells, subendothelial widening, and the presence of platelet thrombi that obstruct the vascular lumen [2]. The laboratory assessment of TMA involves identifying the presence of anemia and thrombocytopenia associated with signs of hemolysis. Hemolysis can be evaluated through higher levels of lactate dehydrogenase (LDH), the presence of schistocytes in peripheral blood smear, decreased haptoglobin levels, or signs of organ damage in the kidney, liver, heart, brain, or gastrointestinal system [3]. Once the diagnosis of TMA is established, a complete workup is necessary to access the underlying condition. TMA can be classified into primary and secondary forms based on its etiology [4]. The primary conditions associated with TMA are linked to two main causes: ADAMTS13 deficiency, leading to Thrombotic Thrombocytopenic Purpura (TTP), and complement dysregulation, leading to complement-mediated TMA (CM-TMA), also named atypical Hemolytic Uremic Syndrome (aHUS) [5]. The pathogenesis of TTP is associated with either acquired (antibodies) or genetic deficiency in ADAMTS13 activity [6]. CM-TMA is linked to genetic variants in complement genes or the presence of antibodies against factor H. Treatment with complement C5 blockade has shown significant improvements in outcomes for CM-TMA patients [7]. ADAMTS13 activity measurement plays a pivotal role in differentiating TTP from other types of TMA, with values falling below 10% serving as a hallmark of TTP. However, ADAMST13 activity measurement results are often delayed and TTP requires immediate treatment. To address this challenge, an algorithm called the PLASMIC score was developed to differentiate between TTP and other forms of TMA. Currently, this scoring system is widely utilized in clinical practice [8].

The secondary TMA conditions encompass a wide array of manifestations that arise as a result of several factors. These factors include malignancy, pregnancy, infections such as viral infection, pneumococcal infection, HIV, and hepatitis, Shiga toxin-producing infections (STEC-SHU), transplantation-associated TMA, malignant hypertension, medications, metabolic defects like cobalamin deficiency, and autoimmune diseases [9].

The differential diagnosis of TMA represents a challenging stage, and certain TMA conditions, such as aHUS, do not have a definitive diagnostic test, which often necessitates an exclusion diagnosis approach [5]. TMA is a life-threatening condition that demands early and timely diagnosis to prevent significant morbidity and mortality. Currently, there is no clinical score to effectively differentiate aHUS from secondary TMA. The primary aim was to develop an algorithm for classifying the thrombotic microangiopathy (TMA), which will be referred to as the TMA-INSIGHT score. The secondary aim was to determine the frequency of TMA phenotypes based on their underlying etiology.

## Methods

This study was a single-center retrospective cohort study that involves hospitalized patients with thrombotic microangiopathy (TMA) at Hospital das Clínicas of Botucatu, UNESP. The inclusion criteria encompassed all consecutive patients diagnosed with TMA between June 1, 2012, and December 31, 2021.

We excluded patients with autoimmune hemolytic anemia, and cases without evidence of organ damage (insufficient criteria for TMA). The study was approved by the Research Ethics Committee of UNESP (number 65300922.0.0000.5411).

### TMA definition

We defined TMA as the presence of anemia (Hb < 10 g/dL) and thrombocytopenia (platelet count < 150,000/µL) associated with signs of hemolysis and organ damage. Hemolysis was characterized by an increase in lactate dehydrogenase (LDH) levels greater than 1.5 times the upper reference limit, reduced haptoglobin levels (below the reference range), or the presence of schistocytes in the peripheral blood smear. We combine those findings with evidence of organ damage defined as kidney damage, central nervous system damage, gastrointestinal damage, cardiovascular damage, or vascular damage.

We defined the kidney damage as an increase in 30% in baseline creatinine [10]. We defined central nervous system damage as altered/reduced conscience and or convulsions. We defined gastrointestinal damage as the presence of diarrhea, pancreatitis, or colitis. Cardiovascular damage was defined as myocardial infarction, or arrythmias. Vascular damage was defined by presence of thrombosis.

### Data extraction

Using the defined criteria for TMA, we retrieved the records of hospitalized patients from the electronic health records using automatic search criteria (supplementary).

### Comorbidities and associated conditions

We define the associated comorbidities and conditions as follow: hypertension, malignant hypertension; diabetes, cardiovascular disease, kidney disease, liver disease, active transplant, active malignance, active pregnancy, autoimmune disease, COVID, active infection, and mean arterial pressure (MAP). The MAP was calculated with the systolic and diastolic pressure at onset (first measure of the hospital admission). Detailed definitions of comorbidities can be found in the supplementary file.

### Laboratory exams

All laboratory exams were retrieved within the course of hospitalization. We retrieved the minimum and maximum values of laboratory exams performed within the hospitalization: creatinine, urea, hemoglobin, hematocrit, platelets, Lactate dehydrogenase (LDH), haptoglobin, Schistocytes, Coombs Test, aspartate aminotransferase (AST), alanine aminotransferase (ALT), Total bilirubin, Prothrombin time, and Partial thromboplastin time (Reference values in the supplementary). The delta creatinine was calculated by dividing the maximum creatinine value by the minimum creatinine value.

We checked for additional parameters as available. Those exams were retrieved in the electronic health record: ADAMTS13 activity, Shiga toxin PCR, stool culture, fundoscopy, complement tests, and genetic analysis.

### TMA phenotype classification

The patients were categorized based on the International Statistical Classification of Diseases and Related Health Problems, 10th Revision (ICD-10), in combination with their medical history, signs of organ damage, and laboratory exam results (Description of the ICD-10 codes in the supplementary). We classified the patients into eight distinct groups based on their TMA phenotype.

1. Secondary to infections: sepsis, other infections, HIV, and COVID-19;
2. Secondary to Malignant Hypertension;
3. Secondary to Transplant;
4. Secondary to Malignancy;
5. Secondary to Pregnancy.
6. Thrombotic Thrombocytopenic Purpura (TTP);
7. Shiga toxin-mediated hemolytic uremic syndrome (STEC-SHU);
8. Complement Mediated TMA (aHUS): defined as TMA for which all other TMA diagnoses were excluded.

### Statistical analysis

The categorical variables were described in number and percentage. The numeric variables were reported in median and percentiles (25 and 75%). To evaluate the differences in TMA laboratory values between groups, we employed the Kruskal-Wallis test. For multiple comparisons between groups, we utilized the Dunn Test with Holm adjustment (post-hoc test). All laboratory TMA values were found to be non-normally distributed and were assessed using the Shapiro Test.

### Predictive model

We imputed the variables with more than 30% missing values using the term “not performed”. The other variables were imputed using nearest neighbors (KNN). To fit the boosted tree models, we used one-hot encoding, and to fit the lasso regression we transformed the numeric predictors using Yeo Johnson approach. To adjust to the class imbalance, the synthetic minority over-sampling (SMOTE) [11] method was used to create synthetic classes in the training set (Balancing).

### Model training

We split the data into derivation (training, 75%) and validation (test, 25%) data set using a random split stratified by the target (TMA classification). We fitted a multinomial classification model using gradient boosting decision trees (XGBoost, LightGBM), and a Lasso regression. In the training data, 10-fold-cross validation was used to select the best hyperparameters of the models aimed to reduce the bias.

### Selection of predictors

In the training set, we applied a Lasso model to select predictors for inclusion in the model training. Specifically, we included in the analysis those predictors that were selected by the Lasso with a regression coefficient greater than zero.

### Assessment of accuracy

The accuracy of the derivation cohort model was tested on the data of the validation cohort. We used the area under the receiver operating characteristic curve (AUC-ROC) and logarithmic loss to discriminate the ability of the models in the train and test set. We used software R version 4.0.2 and the packages tidymodels. A detailed description of machine learning analysis was in the Supplementary.

### TMA-INSIGHT score (Thrombotic Microangiopathy Identification and Scoring for Optimal Guidance)

The TMA-INSIGHT score was determined by selecting the model with higher values of ROC-AUC and accuracy, along with optimal values of sensitivity and specificity. The TMA-INSIGHT score provides the probability of patients being classified into each TMA phenotype.

## Results

**Fig. 1:**
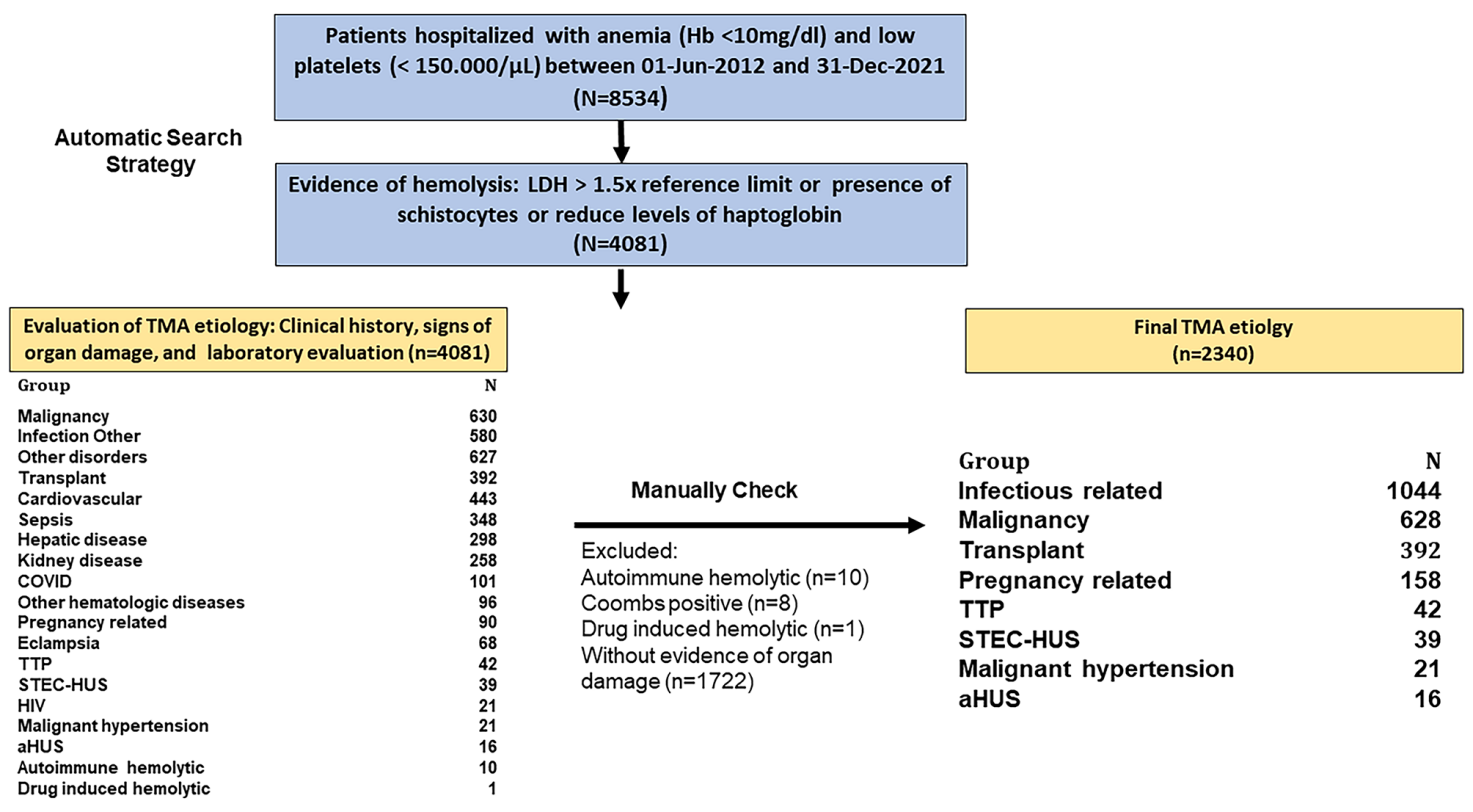
Flowchart Steps to Retrieve Patients with Thrombotic Microangiopathy (TMA)

We identified 8534 patients who met the initial criteria for anemia associated with thrombocytopenia. Upon combining these patients with those showing signs of hemolysis, the cohort was reduced to 4081 patients. The classification process involved assessing the patients based on ICD-10 code diagnostics, medical history, sign of organ damage, and laboratory exams. From the analysis, we excluded patients with drug-induced hemolytic anemia (n = 1), autoimmune hemolytic anemia (n = 10), and those with a positive Coombs test (n = 8). Additionally, patients without evidence of organ damage were excluded (n = 1722). Consequently, the final classification yielded eight phenotypes of TMA, comprising a total of 2340 patients (Fig. 1).

**Fig. 2:**
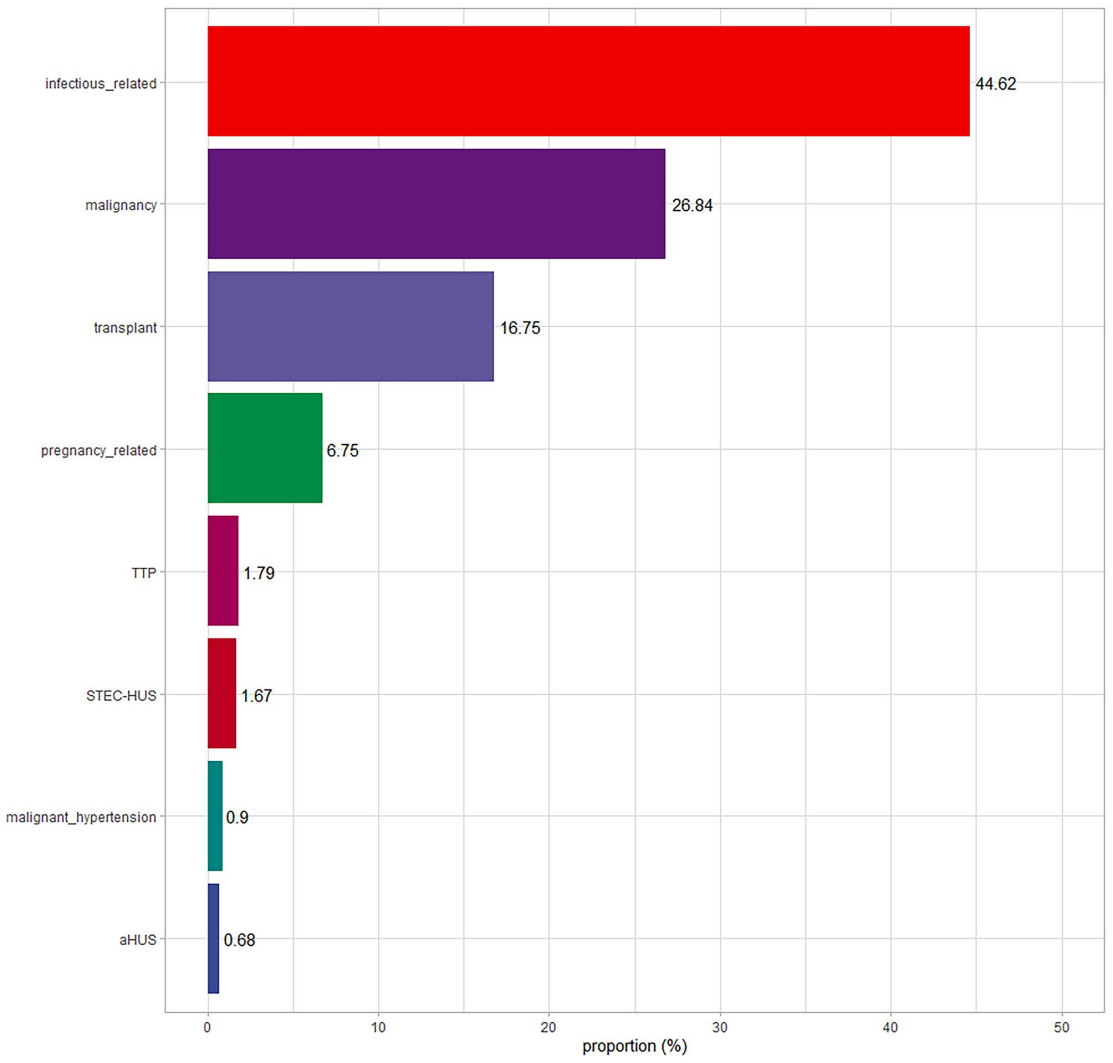
Thrombotic microangiopathy (TMA) by etiology in hospitalized patients

Secondary TMA was identified in 97.5% of the patients, whereas primary TMA, comprising TTP and aHUS cases, was found in 2.47% of the patients (Fig. 2). Among the cases of primary TMA, TTP was present in 1.79% of the patients, with a minimum platelet value of 10 [4–16]/µL, a median delta creatinine of 1.50 (1.24–1.81) mg/dl, and a mean arterial pressure (MAP) of 87 (78–100) mmHg. Plasmapheresis was administered to 40 out of 42 patients, with only 2 cases where it was not used due to concomitant leukemia. The aHUS patients represented 0.68% of the total TMA cases. Among the aHUS patients, the minimum platelet count was 82 (40–111)/µL, the delta creatinine was 3.11 (2.81–7.24) mg/dl, and the mean arterial pressure (MAP) was 107 (82–109) mmHg. Genetic analysis was performed on 7 aHUS patients, with pathogenic variants observed (2 cases related to CFH, 2 related to CFHR1-CFHR3, 1 to CFB, 1 to CFI, and 1 to C3). Furthermore, 14 aHUS patients received treatment with eculizumab.

Among the secondary TMA etiologies, the most frequent were infectious cases (44.62%), with 9.7% of those being attributed to COVID-19. Malignancy accounted for 26.84% of cases, making it the second most common cause. The third most prevalent etiology was TMA secondary to transplantation, representing 16.75% of the cases. TMA related to pregnancy was present in 6.71%, with eclampsia/HELLP syndrome accounting for 43% of those cases. Patients with STEC-HUS comprised 1.67% of the cohort and were confirmed to have positive stool cultures for *Escherichia Coli*. Malignant hypertension accounted for 0.90% of the cases and was confirmed through typical fundoscopic findings, including disc edema, arteriolar constriction, peripapillary flame-shaped hemorrhages, and cotton-wool spots.

**Fig. 3:**
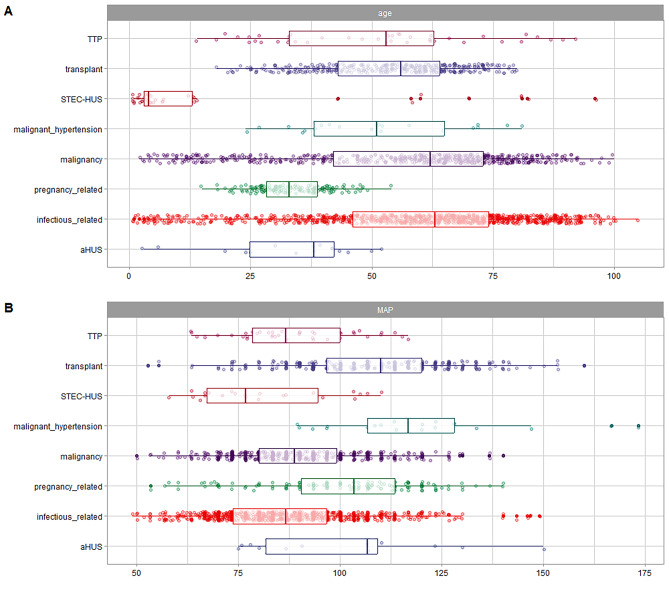
**A**. Age in patients stratified by thrombotic microangiopathy (TMA). **B**. Mean Arterial Pressure (MAP) in patients stratified by thrombotic microangiopathy (TMA).

**Fig. 4:**
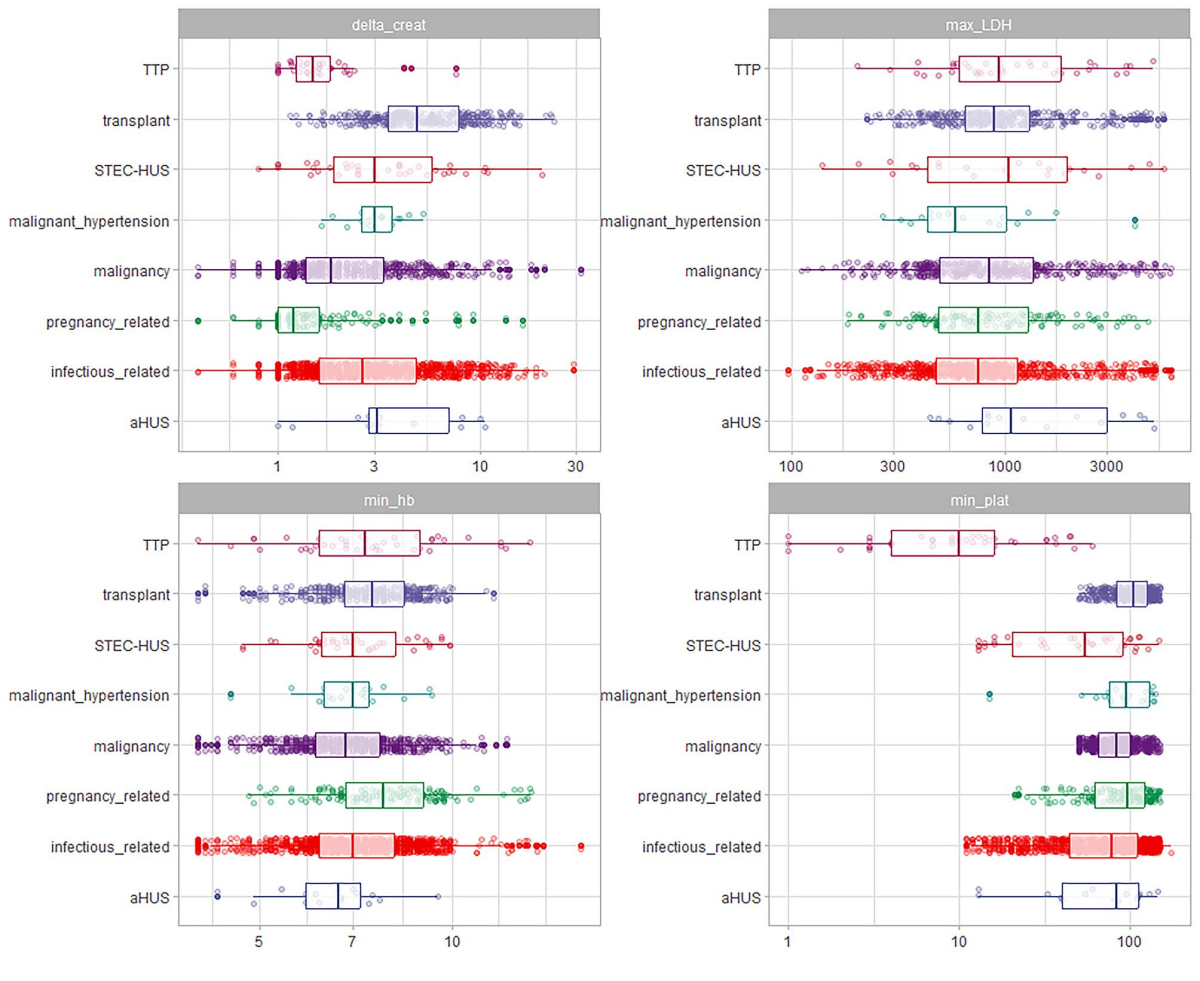
Laboratory exams (delta creatinine, LDH, Hemoglobin, platelets) in patients stratified by thrombotic microangiopathy (TMA). (Values in log10 scale)

**Fig. 5:**
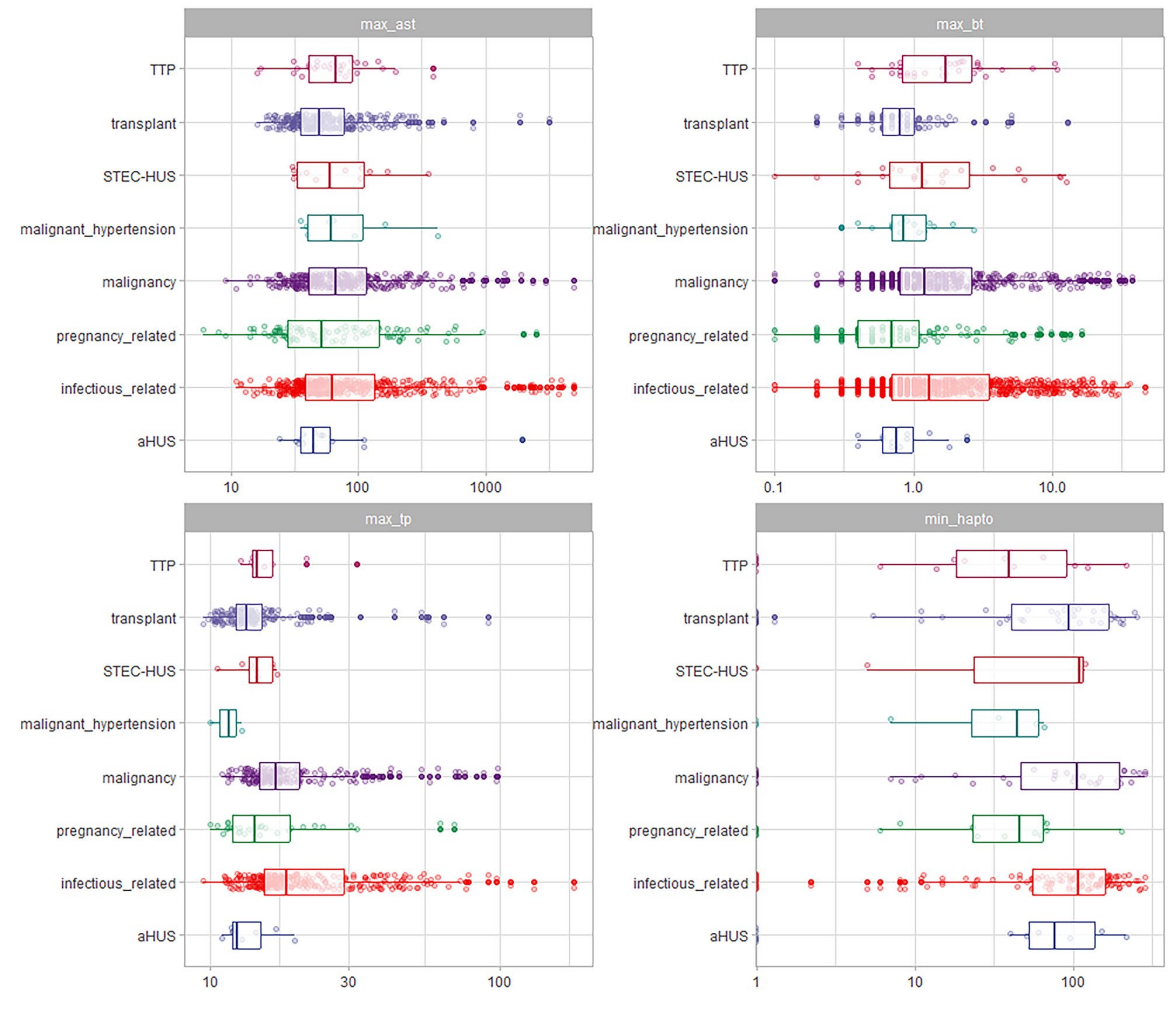
Laboratory exams (AST, Bilirubin, TP and haptoglobin) in patients stratified by thrombotic microangiopathy (TMA). (Values in log10 scale)

In comparison to the other groups, patients with TTP, pregnancy-related TMA, and aHUS were found to have a lower age (Table 1; Fig. 3A, supplementary Table 1). The presence of severe hypertension and cardiovascular disease was more frequent in malignant hypertension. Kidney disease was more commonly found in patients with TMA related to transplantation and malignant hypertension. On the other hand, liver disease was more frequently observed in cases of infectious-related TMA (Table 1). The mean arterial pressure was higher in the groups of patients with malignant hypertension, pregnancy-related TMA, transplant-related TMA, and aHUS (as shown in Fig. 3B and supplementary Table 2). Patients with a transplant had higher levels of delta creatinine compared to the other groups (as shown in supplementary Tables 3 and Fig. 4). Patients with TTP had lower levels of platelets compared to the other groups (as shown in supplementary Table 4). Furthermore, patients with malignancy and infectious-related TMA had higher values of prothrombin time, AST (aspartate aminotransferase) values, and total bilirubin (as demonstrated in Fig. 5). Detailed multiple comparisons between the examinations and TMA classifications can be found in supplementary Tables 4–9. The infectious etiologies associated with TMA were detailed in Supplementary Table 10.

**Table 1:** Baseline characteristics, comorbidities, and associated conditions stratified by thrombotic microangiopathy (TMA) in hospitalized patients

Characteristic	aHUS, N = 16	infectious_related, N = 1,044	pregnancy_related, N = 158	malignancy, N = 628	malignant_hypertension, N = 21	STEC-HUS, N = 39	transplant, N = 392	TTP, N = 42	p-value
age	38 (25, 42)	63 (46, 74)	33 (28, 39)	62 (42, 73)	51 (38, 65)	4 (3, 13)	56 (43, 64)	53 (33, 63)	< 0.001
race									
non-white	3 (19%)	147 (14%)	21 (13%)	66 (11%)	3 (14%)	2 (5.1%)	71 (18%)	7 (17%)	
white	13 (81%)	897 (86%)	137 (87%)	562 (89%)	18 (86%)	37 (95%)	321 (82%)	35 (83%)	
gender									< 0.001
female	9 (56%)	487 (47%)	158 (100%)	286 (46%)	11 (52%)	22 (56%)	175 (45%)	28 (67%)	
male	7 (44%)	557 (53%)	0 (0%)	342 (54%)	10 (48%)	17 (44%)	217 (55%)	14 (33%)	
hypertension	6 (38%)	565 (54%)	33 (21%)	289 (46%)	20 (95%)	8 (21%)	296 (76%)	20 (48%)	< 0.001
hypertension_emergency	0 (0%)	6 (0.6%)	0 (0%)	0 (0%)	21 (100%)	0 (0%)	0 (0%)	0 (0%)	
Unknown	0	0	1	0	0	0	0	0	
diabetes	1 (6.2%)	378 (36%)	5 (3.2%)	185 (29%)	10 (48%)	5 (13%)	133 (34%)	8 (19%)	
cardiovascular_disease	0 (0%)	83 (8.0%)	0 (0%)	21 (3.3%)	6 (29%)	1 (2.6%)	18 (4.6%)	2 (4.8%)	
kidney_disease	6 (38%)	190 (18%)	2 (1.3%)	29 (4.6%)	15 (71%)	2 (5.1%)	282 (72%)	1 (2.4%)	
liver_disease	1 (6.2%)	152 (15%)	2 (1.3%)	42 (6.7%)	3 (14%)	3 (7.7%)	11 (2.8%)	2 (4.8%)	
transplant	14 (88%)	107 (10%)	1 (0.6%)	94 (15%)	6 (29%)	4 (10%)	392 (100%)	2 (4.8%)	
pregnancy	3 (19%)	31 (3.0%)	158 (100%)	13 (2.1%)	0 (0%)	11 (28%)	26 (6.6%)	7 (17%)	
Autoimmune_disease	0 (0%)	58 (5.6%)	2 (1.3%)	7 (1.1%)	2 (9.5%)	3 (7.7%)	16 (4.1%)	6 (14%)	
malignancy	0 (0%)	186 (18%)	1 (0.6%)	628 (100%)	0 (0%)	4 (10%)	6 (1.5%)	6 (14%)	< 0.001
covid	1 (6.2%)	258 (25%)	5 (3.2%)	36 (5.7%)	4 (19%)	3 (7.7%)	22 (5.6%)	1 (2.4%)	
infection	2 (12%)	1,044 (100%)	0 (0%)	0 (0%)	0 (0%)	15 (38%)	0 (0%)	0 (0%)	< 0.001
MAP	107 (82, 109)	87 (74, 97)	103 (90, 113)	89 (80, 99)	117 (107, 128)	77 (67, 94)	110 (97, 120)	87 (78, 100)	< 0.001

### Predictive model

We initially employed a Lasso regression to select all available predictors. This process resulted in a reduced number of predictors used to fit the model, including the following variables: age, delta creatinine, maximum LDH, minimum platelets, minimum hemoglobin, maximum AST, maximum total bilirubin, maximum prothrombin time, maximum partial thromboplastin time, presence of hypertension, presence of cardiovascular disease, presence of kidney disease, presence of liver disease, presence of autoimmune disease, active transplant, active malignancy, active pregnancy, presence of infection, active COVID infection, and mean arterial pressure.

We applied three different models, namely Xgboost, LightGBM, and Lasso regression, to the train set comprising 1754 samples. The performance metrics were then evaluated on the test set containing 586 samples. Among the models, LightGBM demonstrated superior performance, as indicated in Table 2. The LightGBM model achieved an accuracy of 0.979 and a multiclass ROC-AUC of 0.966 (link to the predictive model: https://nephrologymodels.shinyapps.io/TMApred/).

**Table 2:** Performance metrics of machine learning model in the test set to predict thrombotic microangiopathy (TMA) in hospitalized patients

.metric	.estimator	XgBoost	LightGBM	Lasso
accuracy	multiclass	0.9778157	0.9795222	0.9385666
kappa	multiclass	0.9679064	0.9703983	0.9124146
Sensibility	macro	0.8223741	0.8575002	0.7572324
specificity	macro	0.9962477	0.9965055	0.9911797
ppv	macro	0.9120769	0.9340179	0.7446433
npv	macro	0.9969382	0.9971120	0.9897623
mcc	multiclass	0.9680054	0.9704651	0.9127704
j_index	macro	0.8186219	0.8540056	0.7484121
bal_accuracy	macro	0.9093109	0.9270028	0.8742060
detection_prevalence	macro	0.1250000	0.1250000	0.1250000
precision	macro	0.9120769	0.9340179	0.7446433
recall	macro	0.8223741	0.8575002	0.7572324
f_meas	macro	0.8558384	0.8885345	0.7474930

**Fig. 6:**
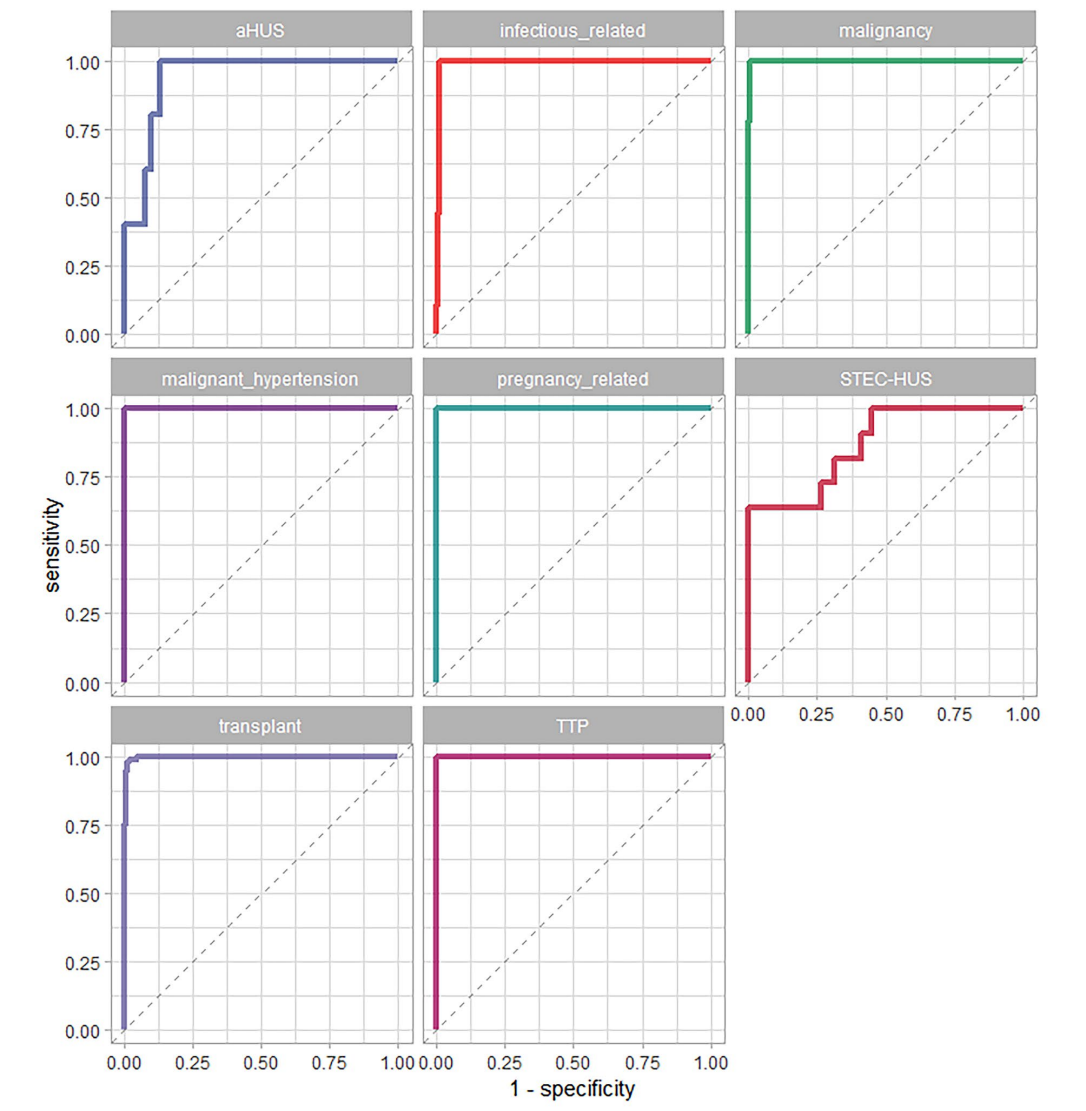
ROC AUC of LightGBM model to predict thrombotic microangiopathy (TMA) stratified by the predictions in each class

The predictions showed higher accuracy in cases of infectious-related TMA, malignancy, malignant hypertension, pregnancy-related TMA, transplant-related TMA, and TTP. However, the predictions had lower confidence in cases of aHUS and STEC-HUS, as shown in Fig. 6.

**Fig. 7:**
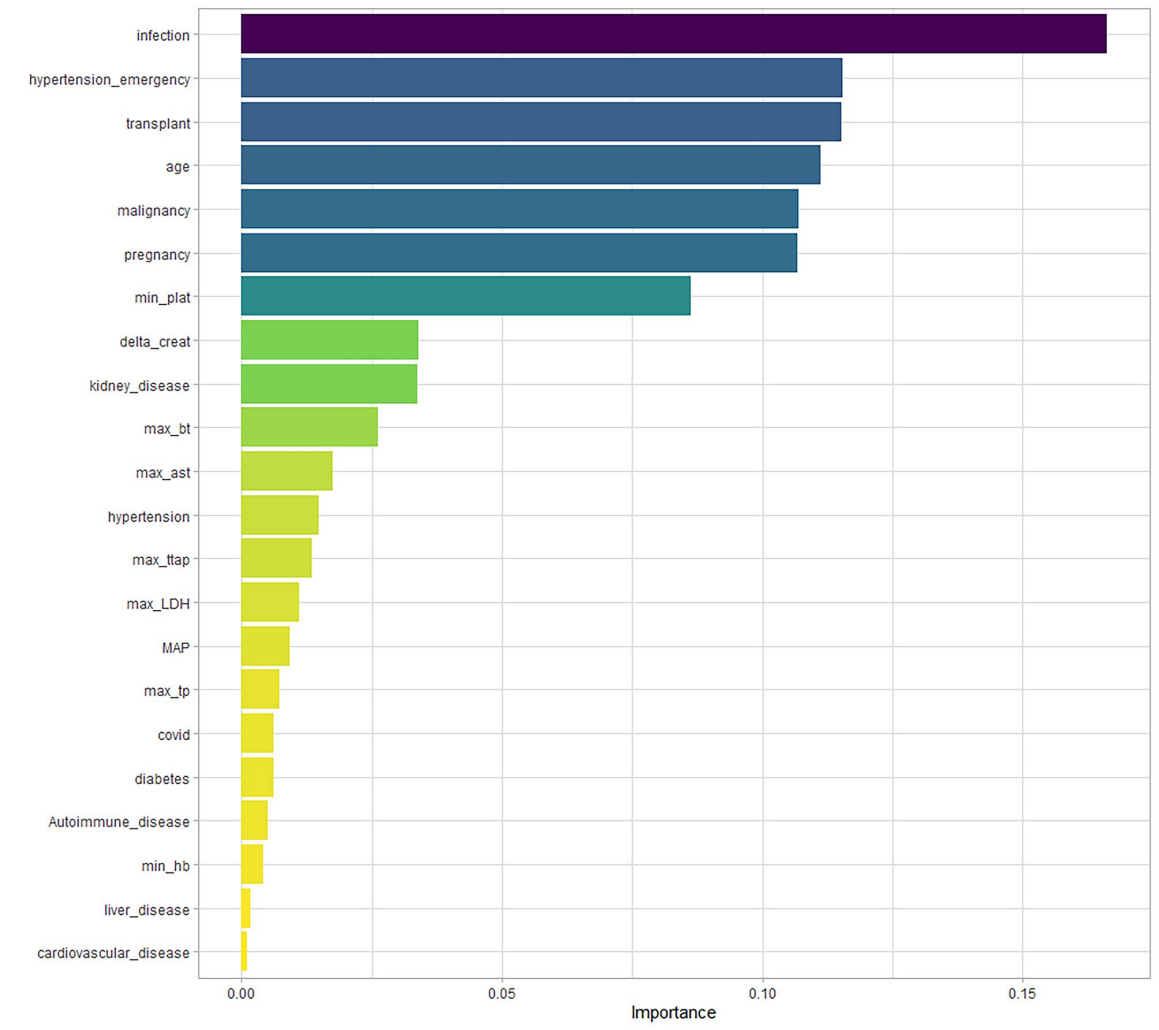
Importance of Predictors of the LightGBM model to predict thrombotic microangiopathy (TMA).

The strongest predictors associated with TMA classification were identified as follows: presence of infection, presence of hypertensive emergency, active transplant, age, active malignancy, active pregnancy, minimum platelets, delta creatinine, kidney disease, and active transplant, as shown in Fig. 7.

### Practical examples of TMA-INSIGHT prediction

We provided examples of eight patients, including their clinical characteristics and biochemical data. The TMA-INSIGHT retrieved the class probability for each patient in their respective classes. For more details on these examples, please refer to the Supplementary Practical Examples and Tables 10 and 11.

### External validation

We conducted an external validation of the TMA-INSIGHT score using a Brazilian cohort of aHUS patients [12]. This cohort compromised 75 aHUS cases, including adults (53.4%) and children with a median age of 20.7 years [12]. Within this cohort, we were able to obtain clinical and biochemical data to validate the TMA-INSIGHT score. The results showed that the majority of the cases were corrected classified as aHUS (n = 44, 59%), followed by transplant-associated cases (n = 12, 16%), malignant hypertension (n = 8, 11%), pregnancy-related (n = 5, 6.7%), STEC-HUS (n = 3, 4%), and TTP (n = 3, 4%). Both infectious-related and malignancy-related cases demonstrated a zero probability. The model exhibited a multiclass accuracy of 0.586, sensitivity of 0.586, and specificity of 0.941.

## Discussion

In this study, we retrospective retrieved of a higher number of thrombotic microangiopathy (TMA) phenotypes using automated strategies applied to electronic health records in almost 10 years. We successfully classified patients into eight distinct TMA phenotypes. Additionally, we developed a predictive score called TMA-INSIGHT, which incorporates clinical features and laboratory exams to calculate the probability of a patient belonging to a specific TMA phenotype. This approach enables early recognition and accurate classification of TMA cases.

We had significant insights when comparing the TMA phenotypes. Patients with STEC-HUS had lower age compared to the other groups consistent with previous reports [13]. TTP patients in this cohort had lower platelet values, which were associated with a lower delta creatinine, corroborating findings from previous reports of French score [14].

The levels of blood pressure are higher in patients with malignant hypertension, transplantation, aHUS, and pregnancy similar to the previous report [15]. Halimi et al. demonstrated that a mean arterial pressure (MAP) above 116mmHg almost completely ruled out the diagnosis of TTP [15].

The aHUS patients in our cohort had lower age at onset, higher MAP values, and elevated levels of LDH. These clinical and laboratory features of aHUS patients were very similar to the those reported in the Brazilian aHUS Registry [12]. However, there are no specific clinical or laboratory features that characterize aHUS patients.

Importantly, the most common etiologies of thrombotic microangiopathy (TMA) were found to be secondary, which is consistent with previous reports where secondary conditions accounted for 94% of cases [16]. Contrarily, at a reference center, when clinically suspected cases of thrombotic microangiopathy (TMA) were examined, atypical hemolytic uremic syndrome (aHUS) emerged as the most commonly diagnosed condition, representing 27% of the cases [17]. The incidence of drug-induced TMA in our cohort was extremely low, at less than 0.1%, and as such, we made the decision to exclude it from our analysis. In the literature, the prevalence of drug-induced TMA showed high variability, ranging from 5.1% [18] to 26% [16].

As of the present, the available TMA predictor scores have been designed to classify patients into binary outcomes, distinguishing between those with thrombotic thrombocytopenic purpura (TTP) and those with other forms of thrombotic microangiopathy (TMA) [8, 19]. Among these scores, the most significant one is PLASMIC, which utilizes logistic regression based on a dataset of 214 patients from the Harvard TMA Research Collaborative database [8]. The PLASMIC score demonstrated excellent accuracy, surpassing 0.90 in both internal and external validation. Similarly, another score called MED-TMA was developed, and it achieved an accuracy of over 0.95. The MED-TMA score was fitted using a machine learning ensemble model on a dataset comprising 319 patients [19]. These impressive accuracy values indicate the strong predictive capabilities of both PLASMIC [8] and MED-TMA [19] in classifying patients with TMA.

Differently, the TMA-INSIGHT score stands out from previous TMA scores due to its ability to calculate the probability of a patient belonging to one of eight distinct TMA phenotypes. With an overall accuracy exceeding 0.90 in nearly all TMA classes. Importantly the accuracy of TMA-INSIGHT was similar to the PLASMIC score [8] in predicting TTP.

We validated the performance of TMA-INSIGHT in an independent external cohort of Brazilian aHUS patients [12]. The model was able to correctly classify the majority of the cases as aHUS in 59%, demonstrating a relatively good sensitivity and excellent specificity, thereby confirming the utility of the model. The absence of specific diagnostic criteria for aHUS, coupled with its low incidence rate [9], presents a challenging scenario for prediction through machine learning. Machine learning models’ accuracy in predicting rare diseases typically falls within a range of 71–99% [20, 21]. Remarkably, TMA-INSIGHT approaches the upper limit of this accuracy spectrum across most TMA categories, although it exhibits lower values of accuracy when predicting aHUS and STEC-HUS.

This study had several limitations. Firstly, due to its single-center nature, the generalizability of the results may be limited. Further external validation of the TMA-INSIGHT score is necessary to improve its reproducibility and reliability. Although it demonstrated promising results and underwent external validation in aHUS patients, confirming its robustness requires validation across other TMA cohorts. Additionally, not all patients classified in the TTP category had their ADAMTS13 values measured. Instead, their classification was based on clinical findings and their response to plasmapheresis.

In conclusion, secondary conditions were the most common etiologies of TMA. We fit a model to predict TMA and define TMA phenotypic characteristics. Importantly, our study stands as the first of its kind to employ a multiclass model for predicting TMA, encompassing an eight-class categorization that accounts for both primary and secondary conditions. This novel approach provides a more comprehensive and detailed understanding of TMA and its diverse clinical presentations, contributing to improved diagnostic accuracy and patient care.

### Electronic supplementary material

Below is the link to the electronic supplementary material.

Supplementary Material 1

## Data Availability

The datasets used and analysed during the current study are available from the corresponding author on reasonable request.
