# Supplementary material for: A comprehensive model for assessing and classifying patients with thrombotic microangiopathy: the TMA-INSIGHT score
Source: Thromb J. 2023 Nov 22;21:119. doi: 10.1186/s12959-023-00564-6 (PMC10664252; doi:10.1186/s12959-023-00564-6)
Supplement: Supplementary file 1 — Supplementary Material 1 [file 12959_2023_564_MOESM1_ESM.docx]

**Supplementary**

**Search Strategy Criteria**

1. The first strategy involved retrieving all electronic health records at Botucatu UNESP between 01-Jun-2012 and 31-Dec-2021 using the following laboratory criteria: hospitalized patients with hemoglobin levels below 10 mg/dl and platelet counts below 150,000/µL.
2. Subsequently, we filtered these records to include only patients showing signs of hemolysis, which were identified by either LDH levels exceeding 1.5 times the reference limit, the presence of schistocytes, or reduced levels of haptoglobin.
3. We obtained relevant data, such as ICD-10 codes, medical history, additional laboratory exams, and signs of organ damage. Additionally, we conducted further investigations, including ADAMTS13 activity testing, Shiga toxin PCR, stool culture, fundoscopy, complement tests, and genetic analysis.

**Comorbidities and Associated conditions**

1. Hypertension: prior hypertension independent of its duration history
2. Malignant Hypertension: admitted in the hospital with emergency hypertension (diastolic blood pressure higher than 120 mmHg associated with acute target organ damage)
3. Diabetes: presence of diabetes type I or II independent of its duration history
4. Cardiovascular disease: presence of congestive heart failure, left ventricular hypertrophy, or ischemic heart disease.
5. Kidney Disease: presence of chronic kidney disease defined by eGFR lower than 60 mL/min.
6. Liver Disease: presence of cirrhosis, encephalopathy, or ascites.
7. Transplant: active history of solid organ or hematological transplant
8. Malignancy: active history of hematological malignancy or Solid tumor
9. Pregnancy: active pregnancy
10. Autoimmune Disease: presence of Systemic Lupus erythematosus (SLE), or other autoimmune disease (vasculitis, vasculitis ANCA associated).
11. COVID: presence of COVID-19 infection
12. Infection: Evidence of sepsis, invasive fungal infection, tuberculosis, or pneumonia. Active gastrointestinal infection was not included in this category.
13. Mean Arterial Pressure: Calculate with the systolic and diastolic pressure at onset The systolic and diastolic pressure correspond to the first measure of the hospital admission.

We considered as Autoimmune Disease: Systemic Lupus Erythematosus (SLE), vasculitis, and ANCA-associated vasculitis. The diagnostic was determined through a combination of clinical evaluation in physician notes and the presence of positive serological tests. Serological tests included the analysis of specific autoantibodies and inflammatory markers. We retrieved the following autoantibodies: Antinuclear Antibody (ANA) Test, Anti-Double Stranded DNA (anti-dsDNA) Antibody Test, Anti-Neutrophil Cytoplasmic Antibody (ANCA) Test, Complement Component Levels, and Rheumatoid Factor (RF) Test.

**Laboratory Exams and Reference values**

Reference values of the minimum and maximum values of laboratory exams performed within the hospitalization.

1. Creatinine: minimum and maximum values. Reference creatinine: lower than 1.04 mg/dL; Reference delta creatinine: lower than 1.3 mg/dL
2. Urea: minimum and maximum values. Reference: lower than 42 mg/dL
3. Hemoglobin: minimum and maximum values. Reference: 13 to 17 g/dL
4. Hematocrit: minimum and maximum values. Reference: 40 to 50%
5. Platelets: minimum and maximum values. Reference: higher than 150.000/µL
6. Lactate dehydrogenase (LDH): minimum and maximum values: Reference range: 120 to 246 U/L
7. Haptoglobin: minimum and maximum values: Reference range: 25 to 140 mg/dL
8. Schistocytes: presence of schistocytes in peripheral blood analysis. Reference: negative
9. Coombs Test: antiglobulin test. Reference: negative
10. AST: minimum and maximum values: Reference range: 14 to 59 U/L
11. ALT: minimum and maximum values: Reference range: lower 50 U/L
12. Total Bilirubin: minimum and maximum values: Reference range: 0.2 to 1.3 mg/dL
13. Prothrombin time: minimum and maximum values: Reference until 14 seconds
14. Partial thromboplastin time: minimum and maximum values: Reference: until 40 seconds

**Description of ICD-10 codes classifications for TMA phenotype classification:**

1. Thrombotic thrombocytopenic purpura (TTP): D69.3; D696. Associated with clinical diagnosis and ADAMTS13 deficiency.
2. Autoimmune Hemolytic anemias: D59.1; D59.9. Associated with positive Coombs test.
3. Drug induced Hemolytic anemias: D59.2. Associated history of use of drug induced hemolytic anemia.
4. Hemolytic Uremic Syndrome: D59.3; Classified according to medical history in STEC-HUS, or aHUS
5. Secondary to STEC-HUS: A00 to A09 and evidence of gastrointestinal bacterial infection (Shiga PCR or stool culture)
6. Secondary to other hematologic diseases: D500 to D582; D600 to D899
7. Secondary to malignant hypertension: Codes N or I associated with urgency or emergency hypertension and presence of altered retinal examination.
8. Secondary to SARS-COV-2: B342.
9. Secondary to HIV: B24
10. Secondary to sepsis: A418 to A419
11. Secondary to other infections: other infections not related to sepsis
12. Secondary to malignancy: C00 to D449
13. Secondary to cardiovascular: I00 to I99
14. Secondary to hepatic disease: K700 to K778
15. Secondary to kidney disease: N170 to N179; N180
16. Secondary to eclampsia: O140 to O159. Clinical diagnosis of preeclampsia, eclampsia, or HELLP syndrome.
17. Other conditions related to gestation: O100 to O998; P00 to P969
18. Secondary to transplantation: Z900 to Z999
19. Secondary to other disorders: other ICD-10 codes

**Detailed information about machine learning analysis**

Machine learning is a subset of artificial intelligence that enables computers to learn from data and make predictions or decisions without being explicitly programmed for a specific task[1]. Machine learning involves the following key steps:

1. **Data Collection:** Gather relevant data that contains features (input variables) and the target variable. We used in the present work a database of a tertiary hospital with TMA patients.

2. **Data Preprocessing:** Clean and preprocess the data by handling missing values, outliers, and scaling the features to ensure they are on a consistent scale. We used the steps described in the predictive model (KNN imputation, Yeo Johnson approach). We also applied the SMOTE algorithm. SMOTE (Synthetic Minority Over-sampling Technique) is an algorithm used in the field of imbalanced classification to address the problem of having significantly more examples of one class (the majority class) than another (the minority class). It works by generating synthetic samples for the minority class to balance the class distribution.

3. **Feature Selection/Engineering:** Choose relevant features or create new ones to improve model performance. This step can significantly impact the model's accuracy. We used a Lasso model to perform feature selection.

4. **Model Selection:** Choose an appropriate machine learning algorithm or model based on the problem type (classification, regression, clustering) and the characteristics of the data. We fitted three different models: Lasso regression, XgBoost, and LightGBM.

5. **Model Training:** Use a portion of the data (training set) to train the chosen model. During training, the model learns patterns and relationships in the data. We used a random split of 75% of the data to train the model.

6. **Model Evaluation:** Assess the model's performance using a separate dataset (validation or test set) that it has never seen before. Common evaluation metrics include accuracy, precision, recall, F1-score, and mean squared error, depending on the problem type. We verify the accuracy of the model in the test set using ROC-AUC and logarithmic loss.

7. **Hyperparameter Tuning:** Adjust the model's hyperparameters (settings that control the learning process) to optimize its performance. We performed hyperparameter tuning in the train set through 10-fold cross-validation.

8. **Model Deployment:** Deploy it in a real-world application to make predictions on new, unseen data. We provided an application to use the model.

**Description of XGBoost, LightGBM, and Lasso:**

1. **XGBoost (Extreme Gradient Boosting):** XGBoost is a powerful and popular gradient boosting algorithm used for both regression and classification tasks. It works by combining the predictions of multiple weak learners (typically decision trees) to create a strong ensemble model. XGBoost is known for its efficiency, speed, and ability to handle large datasets. It also includes regularization techniques to prevent overfitting[2].

2. **LightGBM:** LightGBM is another gradient boosting framework that is designed to be faster and more memory-efficient than traditional gradient boosting methods like XGBoost. It uses a histogram-based approach to split data during training, which reduces the time and memory required. LightGBM is particularly well-suited for large datasets and is known for its high performance[3].

3. **Lasso Model (Least Absolute Shrinkage and Selection Operator):** Lasso is a linear regression model with a regularization technique called L1 regularization. Lasso adds a penalty term to the linear regression loss function, which encourages the model to shrink the coefficients of less important features to zero. This feature selection property makes Lasso useful for identifying and selecting the most relevant features in a dataset, which can help improve model interpretability and reduce overfitting.

**References**

1. Galuzio PP, Cherif A. Recent Advances and Future Perspectives in the Use of Machine Learning and Mathematical Models in Nephrology. Adv Chronic Kidney Dis. 2022;29:472–9.

2. Friedman JH. Greedy Function Approximation: A Gradient Boosting Machine. The Annals of Statistics. 2001;29:1189–232.

3. Ke G, Meng Q, Finley T, Wang T, Chen W, Ma W, et al. LightGBM: A Highly Efficient Gradient Boosting Decision Tree. Advances in Neural Information Processing Systems [Internet]. Curran Associates, Inc.; 2017 [cited 2023 Sep 19]. Available from: https://proceedings.neurips.cc/paper/2017/hash/6449f44a102fde848669bdd9eb6b76fa-Abstract.html

**Table 01. Multiple comparisons between age at onset and thrombotic microangiopathy (TMA) groups in hospitalized patients.**

| **.y.** | **group1** | **group2** | **n1** | **n2** | **statistic** | **p** | **p.adj** | **p.adj.signif** |
| --- | --- | --- | --- | --- | --- | --- | --- | --- |
| age | aHUS | infectious_related | 16 | 1044 | 4.819 | 0.000 | 0.000 | **** |
| age | aHUS | pregnancy_related | 16 | 158 | -0.182 | 0.856 | 1.000 | ns |
| age | aHUS | malignancy | 16 | 628 | 4.446 | 0.000 | 0.000 | *** |
| age | aHUS | malignant_hypertension | 16 | 21 | 2.301 | 0.021 | 0.235 | ns |
| age | aHUS | STEC-HUS | 16 | 39 | -0.552 | 0.581 | 1.000 | ns |
| age | aHUS | transplant | 16 | 392 | 3.425 | 0.001 | 0.009 | ** |
| age | aHUS | TTP | 16 | 42 | 2.582 | 0.010 | 0.118 | ns |
| age | infectious_related | pregnancy_related | 1044 | 158 | -14.781 | 0.000 | 0.000 | **** |
| age | infectious_related | malignancy | 1044 | 628 | -1.754 | 0.079 | 0.635 | ns |
| age | infectious_related | malignant_hypertension | 1044 | 21 | -2.043 | 0.041 | 0.369 | ns |
| age | infectious_related | STEC-HUS | 1044 | 39 | -8.449 | 0.000 | 0.000 | **** |
| age | infectious_related | transplant | 1044 | 392 | -5.750 | 0.000 | 0.000 | **** |
| age | infectious_related | TTP | 1044 | 42 | -2.894 | 0.004 | 0.049 | * |
| age | pregnancy_related | malignancy | 158 | 628 | 13.182 | 0.000 | 0.000 | **** |
| age | pregnancy_related | malignant_hypertension | 158 | 21 | 3.493 | 0.000 | 0.008 | ** |
| age | pregnancy_related | STEC-HUS | 158 | 39 | -0.650 | 0.516 | 1.000 | ns |
| age | pregnancy_related | transplant | 158 | 392 | 9.775 | 0.000 | 0.000 | **** |
| age | pregnancy_related | TTP | 158 | 42 | 4.644 | 0.000 | 0.000 | **** |
| age | malignancy | malignant_hypertension | 628 | 21 | -1.631 | 0.103 | 0.720 | ns |
| age | malignancy | STEC-HUS | 628 | 39 | -7.813 | 0.000 | 0.000 | **** |
| age | malignancy | transplant | 628 | 392 | -3.915 | 0.000 | 0.002 | ** |
| age | malignancy | TTP | 628 | 42 | -2.302 | 0.021 | 0.235 | ns |
| age | malignant_hypertension | STEC-HUS | 21 | 39 | -3.427 | 0.001 | 0.009 | ** |
| age | malignant_hypertension | transplant | 21 | 392 | 0.490 | 0.624 | 1.000 | ns |
| age | malignant_hypertension | TTP | 21 | 42 | -0.019 | 0.985 | 1.000 | ns |
| age | STEC-HUS | transplant | 39 | 392 | 6.178 | 0.000 | 0.000 | **** |
| age | STEC-HUS | TTP | 39 | 42 | 4.148 | 0.000 | 0.001 | *** |
| age | transplant | TTP | 392 | 42 | -0.708 | 0.479 | 1.000 | ns |

Global Kruskal-Wallis rank sum test, p <0.001

Dunn Test adjustment to multiple comparisons with Holm (post-hoc test)

**Table 02. Multiple comparisons between mean arterial pressure (MAP) and thrombotic microangiopathy (TMA) groups in hospitalized patients.**

| **.y.** | **group1** | **group2** | **n1** | **n2** | **statistic** | **p** | **p.adj** | **p.adj.signif** |
| --- | --- | --- | --- | --- | --- | --- | --- | --- |
| MAP | aHUS | infectious_related | 14 | 994 | -2.593 | 0.010 | 0.114 | ns |
| MAP | aHUS | pregnancy_related | 14 | 157 | 0.320 | 0.749 | 1.000 | ns |
| MAP | aHUS | malignancy | 14 | 579 | -1.982 | 0.048 | 0.428 | ns |
| MAP | aHUS | malignant_hypertension | 14 | 21 | 2.127 | 0.033 | 0.334 | ns |
| MAP | aHUS | STEC-HUS | 14 | 23 | -2.992 | 0.003 | 0.039 | * |
| MAP | aHUS | transplant | 14 | 375 | 1.490 | 0.136 | 0.802 | ns |
| MAP | aHUS | TTP | 14 | 41 | -1.889 | 0.059 | 0.471 | ns |
| MAP | infectious_related | pregnancy_related | 994 | 157 | 9.167 | 0.000 | 0.000 | **** |
| MAP | infectious_related | malignancy | 994 | 579 | 3.099 | 0.002 | 0.029 | * |
| MAP | infectious_related | malignant_hypertension | 994 | 21 | 6.494 | 0.000 | 0.000 | **** |
| MAP | infectious_related | STEC-HUS | 994 | 23 | -1.500 | 0.134 | 0.802 | ns |
| MAP | infectious_related | transplant | 994 | 375 | 18.212 | 0.000 | 0.000 | **** |
| MAP | infectious_related | TTP | 994 | 41 | 0.711 | 0.477 | 1.000 | ns |
| MAP | pregnancy_related | malignancy | 157 | 579 | -6.949 | 0.000 | 0.000 | **** |
| MAP | pregnancy_related | malignant_hypertension | 157 | 21 | 2.775 | 0.006 | 0.072 | ns |
| MAP | pregnancy_related | STEC-HUS | 157 | 23 | -4.943 | 0.000 | 0.000 | **** |
| MAP | pregnancy_related | transplant | 157 | 375 | 3.329 | 0.001 | 0.014 | * |
| MAP | pregnancy_related | TTP | 157 | 41 | -3.842 | 0.000 | 0.002 | ** |
| MAP | malignancy | malignant_hypertension | 579 | 21 | 5.717 | 0.000 | 0.000 | **** |
| MAP | malignancy | STEC-HUS | 579 | 23 | -2.250 | 0.024 | 0.269 | ns |
| MAP | malignancy | transplant | 579 | 375 | 14.207 | 0.000 | 0.000 | **** |
| MAP | malignancy | TTP | 579 | 41 | -0.301 | 0.763 | 1.000 | ns |
| MAP | malignant_hypertension | STEC-HUS | 21 | 23 | -5.792 | 0.000 | 0.000 | **** |
| MAP | malignant_hypertension | transplant | 21 | 375 | -1.464 | 0.143 | 0.802 | ns |
| MAP | malignant_hypertension | TTP | 21 | 41 | -4.914 | 0.000 | 0.000 | **** |
| MAP | STEC-HUS | transplant | 23 | 375 | 6.611 | 0.000 | 0.000 | **** |
| MAP | STEC-HUS | TTP | 23 | 41 | 1.649 | 0.099 | 0.693 | ns |
| MAP | transplant | TTP | 375 | 41 | -6.021 | 0.000 | 0.000 | **** |

Global Kruskal-Wallis rank sum test, p <0.001

Dunn Test adjustment to multiple comparisons with Holm (post-hoc test)

**Table 03. Laboratory exams stratified by thrombotic microangiopathy (TMA) in hospitalized patients.**

| **Characteristic** | **aHUS**, N = 16 | **infectious_related**, N = 1,044 | **pregnancy_related**, N = 158 | **malignancy**, N = 628 | **malignant_hypertension**, N = 21 | **STEC-HUS**, N = 39 | **transplant**, N = 392 | **TTP**, N = 42 | **p-value** |
| --- | --- | --- | --- | --- | --- | --- | --- | --- | --- |
| min_creat | 1.60 (1.18, 2.17) | 0.60 (0.50, 1.10) | 0.60 (0.50, 0.70) | 0.50 (0.50, 0.70) | 2.20 (1.25, 4.32) | 0.40 (0.20, 0.60) | 2.00 (1.20, 3.20) | 0.60 (0.50, 0.70) | <0.001 |
| Unknown | 0 | 6 | 8 | 11 | 1 | 0 | 0 | 2 |  |
| min_urea | 36 (32, 52) | 32 (18, 54) | 21 (17, 28) | 21 (12, 33) | 60 (37, 77) | 15 (9, 32) | 52 (35, 71) | 28 (21, 38) | <0.001 |
| Unknown | 0 | 7 | 9 | 10 | 1 | 0 | 0 | 2 |  |
| min_hb | 6.65 (5.90, 7.17) | 7.00 (6.20, 8.10) | 7.80 (6.80, 9.00) | 6.80 (6.10, 7.70) | 7.00 (6.30, 7.40) | 7.00 (6.25, 8.15) | 7.50 (6.77, 8.40) | 7.30 (6.20, 8.90) | <0.001 |
| Unknown | 0 | 2 | 0 | 2 | 0 | 0 | 0 | 1 |  |
| min_ht | 20.6 (17.5, 21.6) | 21.7 (19.2, 25.0) | 24.0 (21.2, 27.5) | 20.9 (18.4, 23.7) | 21.7 (19.8, 23.5) | 22.1 (19.9, 25.5) | 23.0 (20.6, 25.7) | 21.9 (19.4, 27.5) | <0.001 |
| Unknown | 0 | 2 | 0 | 2 | 0 | 0 | 0 | 1 |  |
| min_plat | 82 (40, 111) | 78 (44, 110) | 96 (62, 121) | 82 (65, 100) | 94 (75, 128) | 54 (20, 90) | 104 (83, 125) | 10 (4, 16) | <0.001 |
| Unknown | 0 | 3 | 0 | 4 | 0 | 0 | 1 | 1 |  |
| min_LDH | 351 (259, 440) | 458 (274, 735) | 551 (374, 854) | 404 (235, 644) | 401 (265, 654) | 496 (244, 988) | 418 (241, 540) | 446 (300, 614) | <0.001 |
| Unknown | 0 | 269 | 11 | 134 | 5 | 17 | 4 | 3 |  |
| min_ast | 15 (12, 20) | 28 (19, 43) | 31 (22, 62) | 24 (16, 36) | 44 (31, 53) | 25 (19, 30) | 21 (16, 27) | 25 (18, 32) | <0.001 |
| Unknown | 2 | 584 | 53 | 287 | 13 | 27 | 96 | 14 |  |
| min_alt | 12 (8, 21) | 22 (14, 38) | 17 (11, 28) | 17 (12, 26) | 17 (12, 32) | 21 (14, 30) | 13 (9, 20) | 27 (17, 33) | <0.001 |
| Unknown | 10 | 622 | 110 | 417 | 13 | 20 | 267 | 33 |  |
| min_bt | 0.35 (0.28, 0.53) | 0.60 (0.40, 1.05) | 0.40 (0.20, 0.50) | 0.50 (0.30, 0.90) | 0.70 (0.40, 1.00) | 0.65 (0.30, 1.05) | 0.50 (0.40, 0.70) | 0.60 (0.40, 0.78) | <0.001 |
| Unknown | 4 | 185 | 15 | 130 | 5 | 15 | 290 | 8 |  |
| min_hapto | 20 (0, 69) | 70 (0, 144) | 8 (0, 57) | 76 (0, 146) | 7 (0, 46) | 5 (0, 110) | 47 (0, 132) | 14 (0, 42) | 0.021 |
| Unknown | 4 | 888 | 137 | 585 | 14 | 34 | 343 | 25 |  |
| min_tp | 11.9 (11.5, 14.0) | 14.9 (13.1, 17.0) | 13.3 (11.4, 14.8) | 14.3 (13.1, 16.1) | 11.6 (10.8, 12.2) | 14.1 (12.9, 14.9) | 12.8 (11.5, 13.7) | 12.6 (11.0, 13.9) | <0.001 |
| Unknown | 8 | 754 | 127 | 424 | 18 | 31 | 215 | 33 |  |
| min_ttap | 35 (29, 37) | 33 (27, 41) | 30 (26, 34) | 32 (27, 40) | 31 (29, 33) | 39 (28, 45) | 33 (27, 38) | 24 (22, 24) | 0.016 |
| Unknown | 8 | 760 | 127 | 427 | 18 | 31 | 216 | 32 |  |
| max_creat | 6.7 (4.5, 10.9) | 2.3 (1.1, 4.4) | 0.8 (0.6, 1.1) | 1.2 (0.8, 2.4) | 8.0 (4.0, 10.5) | 1.1 (0.8, 2.0) | 10.2 (8.8, 12.1) | 0.9 (0.8, 1.2) | <0.001 |
| Unknown | 2 | 11 | 8 | 12 | 4 | 0 | 20 | 2 |  |
| max_urea | 163 (113, 190) | 142 (74, 216) | 38 (24, 50) | 72 (44, 123) | 202 (144, 239) | 104 (65, 146) | 189 (157, 220) | 62 (38, 100) | <0.001 |
| Unknown | 0 | 7 | 9 | 10 | 1 | 0 | 0 | 2 |  |
| max_hb | 11.10 (10.57, 12.43) | 10.70 (9.70, 12.00) | 10.55 (9.25, 11.78) | 10.80 (9.70, 12.10) | 10.70 (10.10, 11.90) | 12.20 (10.60, 14.95) | 11.60 (10.67, 13.00) | 11.00 (10.10, 12.10) | <0.001 |
| Unknown | 0 | 2 | 0 | 2 | 0 | 0 | 0 | 1 |  |
| max_ht | 33.5 (32.8, 37.6) | 32.7 (29.7, 37.0) | 31.8 (28.2, 36.3) | 32.7 (29.3, 36.6) | 33.3 (30.1, 36.6) | 35.8 (31.8, 44.3) | 35.5 (32.4, 39.3) | 33.6 (31.4, 37.1) | <0.001 |
| Unknown | 0 | 3 | 0 | 3 | 0 | 1 | 0 | 1 |  |
| max_plat | 303 (261, 348) | 211 (142, 301) | 162 (131, 210) | 176 (100, 280) | 260 (216, 305) | 272 (126, 345) | 265 (210, 330) | 146 (56, 239) | <0.001 |
| Unknown | 1 | 121 | 4 | 56 | 0 | 11 | 15 | 4 |  |
| max_LDH | 1,072 (783, 2,990) | 749 (470, 1,140) | 745 (485, 1,282) | 840 (491, 1,346) | 582 (433, 1,014) | 1,037 (432, 1,941) | 884 (648, 1,285) | 930 (604, 1,811) | <0.001 |
| Unknown | 0 | 294 | 15 | 158 | 5 | 16 | 11 | 4 |  |
| max_ast | 44 (36, 60) | 63 (38, 135) | 51 (28, 145) | 66 (41, 116) | 61 (40, 111) | 62 (33, 110) | 49 (35, 76) | 66 (41, 90) | <0.001 |
| Unknown | 2 | 584 | 53 | 287 | 13 | 27 | 96 | 14 |  |
| max_alt | 32 (10, 85) | 61 (26, 134) | 28 (16, 80) | 53 (26, 111) | 32 (23, 102) | 76 (28, 376) | 36 (20, 70) | 51 (27, 84) | <0.001 |
| Unknown | 10 | 622 | 110 | 417 | 13 | 20 | 267 | 33 |  |
| max_bt | 0.8 (0.6, 1.0) | 1.3 (0.7, 3.5) | 0.7 (0.4, 1.1) | 1.2 (0.8, 2.6) | 0.9 (0.7, 1.2) | 1.2 (0.7, 2.6) | 0.8 (0.6, 1.0) | 1.7 (0.8, 2.6) | <0.001 |
| Unknown | 4 | 185 | 15 | 130 | 5 | 15 | 290 | 8 |  |
| max_hapto | 93 (43, 119) | 91 (8, 158) | 14 (0, 68) | 76 (5, 146) | 7 (0, 46) | 5 (5, 110) | 86 (28, 145) | 42 (26, 108) | 0.059 |
| Unknown | 4 | 888 | 137 | 585 | 14 | 34 | 343 | 25 |  |
| max_tp | 12 (12, 15) | 18 (15, 29) | 14 (12, 19) | 17 (15, 20) | 12 (11, 12) | 14 (14, 16) | 13 (12, 15) | 14 (14, 16) | <0.001 |
| Unknown | 8 | 754 | 127 | 424 | 18 | 31 | 215 | 33 |  |
| max_ttap | 35 (29, 41) | 44 (35, 60) | 35 (27, 41) | 40 (32, 52) | 31 (29, 33) | 41 (36, 48) | 37 (31, 44) | 33 (26, 44) | <0.001 |
| Unknown | 8 | 760 | 127 | 427 | 18 | 31 | 216 | 32 |  |
| c3 | 79 (72, 101) | 91 (69, 122) | 124 (82, 134) | 122 (88, 136) | 92 (75, 105) | 96 (75, 147) | 97 (76, 109) | 75 (69, 104) | 0.6 |
| Unknown | 8 | 973 | 151 | 621 | 16 | 36 | 368 | 31 |  |
| c4 | 29 (21, 35) | 29 (18, 37) | 27 (14, 37) | 32 (28, 41) | 31 (24, 50) | 13 (11, 21) | 30 (23, 39) | 12 (8, 17) | 0.002 |
| Unknown | 8 | 974 | 151 | 621 | 16 | 36 | 368 | 30 |  |

**Table 04. Multiple comparisons between minimum platelet and thrombotic microangiopathy (TMA) groups in hospitalized patients.**

| **.y.** | **group1** | **group2** | **n1** | **n2** | **statistic** | **p** | **p.adj** | **p.adj.signif** |
| --- | --- | --- | --- | --- | --- | --- | --- | --- |
| min_plat | aHUS | infectious_related | 16 | 1041 | -0.019 | 0.985 | 1.000 | ns |
| min_plat | aHUS | pregnancy_related | 16 | 158 | 1.456 | 0.145 | 0.727 | ns |
| min_plat | aHUS | malignancy | 16 | 624 | 0.688 | 0.492 | 1.000 | ns |
| min_plat | aHUS | malignant_hypertension | 16 | 21 | 1.621 | 0.105 | 0.707 | ns |
| min_plat | aHUS | STEC-HUS | 16 | 39 | -1.717 | 0.086 | 0.687 | ns |
| min_plat | aHUS | transplant | 16 | 391 | 2.728 | 0.006 | 0.070 | ns |
| min_plat | aHUS | TTP | 16 | 41 | -4.883 | 0.000 | 0.000 | **** |
| min_plat | infectious_related | pregnancy_related | 1041 | 158 | 4.531 | 0.000 | 0.000 | *** |
| min_plat | infectious_related | malignancy | 1041 | 624 | 3.534 | 0.000 | 0.006 | ** |
| min_plat | infectious_related | malignant_hypertension | 1041 | 21 | 2.463 | 0.014 | 0.138 | ns |
| min_plat | infectious_related | STEC-HUS | 1041 | 39 | -3.097 | 0.002 | 0.023 | * |
| min_plat | infectious_related | transplant | 1041 | 391 | 11.812 | 0.000 | 0.000 | **** |
| min_plat | infectious_related | TTP | 1041 | 41 | -9.009 | 0.000 | 0.000 | **** |
| min_plat | pregnancy_related | malignancy | 158 | 624 | -2.335 | 0.020 | 0.176 | ns |
| min_plat | pregnancy_related | malignant_hypertension | 158 | 21 | 0.672 | 0.502 | 1.000 | ns |
| min_plat | pregnancy_related | STEC-HUS | 158 | 39 | -4.988 | 0.000 | 0.000 | **** |
| min_plat | pregnancy_related | transplant | 158 | 391 | 3.328 | 0.001 | 0.011 | * |
| min_plat | pregnancy_related | TTP | 158 | 41 | -10.391 | 0.000 | 0.000 | **** |
| min_plat | malignancy | malignant_hypertension | 624 | 21 | 1.640 | 0.101 | 0.707 | ns |
| min_plat | malignancy | STEC-HUS | 624 | 39 | -4.144 | 0.000 | 0.001 | *** |
| min_plat | malignancy | transplant | 624 | 391 | 8.089 | 0.000 | 0.000 | **** |
| min_plat | malignancy | TTP | 624 | 41 | -10.007 | 0.000 | 0.000 | **** |
| min_plat | malignant_hypertension | STEC-HUS | 21 | 39 | -3.871 | 0.000 | 0.002 | ** |
| min_plat | malignant_hypertension | transplant | 21 | 391 | 0.704 | 0.481 | 1.000 | ns |
| min_plat | malignant_hypertension | TTP | 21 | 41 | -7.368 | 0.000 | 0.000 | **** |
| min_plat | STEC-HUS | transplant | 39 | 391 | 7.180 | 0.000 | 0.000 | **** |
| min_plat | STEC-HUS | TTP | 39 | 41 | -4.155 | 0.000 | 0.001 | *** |
| min_plat | transplant | TTP | 391 | 41 | -13.006 | 0.000 | 0.000 | **** |

Global Kruskal-Wallis rank sum test, p <0.001

Dunn Test adjustment to multiple comparisons with Holm (post-hoc test)

**Table 05. Multiple comparisons between maximum LDH and thrombotic microangiopathy (TMA) groups in hospitalized patients.**

| **.y.** | **group1** | **group2** | **n1** | **n2** | **statistic** | **p** | **p.adj** | **p.adj.signif** |
| --- | --- | --- | --- | --- | --- | --- | --- | --- |
| max_LDH | aHUS | infectious_related | 16 | 750 | -2.560 | 0.010 | 0.272 | ns |
| max_LDH | aHUS | pregnancy_related | 16 | 143 | -2.100 | 0.036 | 0.823 | ns |
| max_LDH | aHUS | malignancy | 16 | 470 | -1.933 | 0.053 | 1.000 | ns |
| max_LDH | aHUS | malignant_hypertension | 16 | 16 | -2.333 | 0.020 | 0.492 | ns |
| max_LDH | aHUS | STEC-HUS | 16 | 23 | -1.140 | 0.254 | 1.000 | ns |
| max_LDH | aHUS | transplant | 16 | 381 | -1.364 | 0.173 | 1.000 | ns |
| max_LDH | aHUS | TTP | 16 | 38 | -0.903 | 0.367 | 1.000 | ns |
| max_LDH | infectious_related | pregnancy_related | 750 | 143 | 1.023 | 0.306 | 1.000 | ns |
| max_LDH | infectious_related | malignancy | 750 | 470 | 2.640 | 0.008 | 0.224 | ns |
| max_LDH | infectious_related | malignant_hypertension | 750 | 16 | -0.704 | 0.481 | 1.000 | ns |
| max_LDH | infectious_related | STEC-HUS | 750 | 23 | 1.302 | 0.193 | 1.000 | ns |
| max_LDH | infectious_related | transplant | 750 | 381 | 4.748 | 0.000 | 0.000 | **** |
| max_LDH | infectious_related | TTP | 750 | 38 | 2.272 | 0.023 | 0.554 | ns |
| max_LDH | pregnancy_related | malignancy | 143 | 470 | 0.649 | 0.516 | 1.000 | ns |
| max_LDH | pregnancy_related | malignant_hypertension | 143 | 16 | -1.029 | 0.303 | 1.000 | ns |
| max_LDH | pregnancy_related | STEC-HUS | 143 | 23 | 0.811 | 0.417 | 1.000 | ns |
| max_LDH | pregnancy_related | transplant | 143 | 381 | 2.094 | 0.036 | 0.823 | ns |
| max_LDH | pregnancy_related | TTP | 143 | 38 | 1.559 | 0.119 | 1.000 | ns |
| max_LDH | malignancy | malignant_hypertension | 470 | 16 | -1.311 | 0.190 | 1.000 | ns |
| max_LDH | malignancy | STEC-HUS | 470 | 23 | 0.563 | 0.573 | 1.000 | ns |
| max_LDH | malignancy | transplant | 470 | 381 | 2.080 | 0.038 | 0.823 | ns |
| max_LDH | malignancy | TTP | 470 | 38 | 1.319 | 0.187 | 1.000 | ns |
| max_LDH | malignant_hypertension | STEC-HUS | 16 | 23 | 1.393 | 0.164 | 1.000 | ns |
| max_LDH | malignant_hypertension | transplant | 16 | 381 | 1.868 | 0.062 | 1.000 | ns |
| max_LDH | malignant_hypertension | TTP | 16 | 38 | 1.865 | 0.062 | 1.000 | ns |
| max_LDH | STEC-HUS | transplant | 23 | 381 | 0.108 | 0.914 | 1.000 | ns |
| max_LDH | STEC-HUS | TTP | 23 | 38 | 0.387 | 0.699 | 1.000 | ns |
| max_LDH | transplant | TTP | 381 | 38 | 0.465 | 0.642 | 1.000 | ns |

Global Kruskal-Wallis rank sum test, p <0.001

Dunn Test adjustment to multiple comparisons with Holm (post-hoc test)

**Table 06. Multiple comparisons between minimum hemoglobin and thrombotic microangiopathy (TMA) groups in hospitalized patients.**

| **.y.** | **group1** | **group2** | **n1** | **n2** | **statistic** | **p** | **p.adj** | **p.adj.signif** |
| --- | --- | --- | --- | --- | --- | --- | --- | --- |
| min_hb | aHUS | infectious_related | 16 | 1042 | 1.703 | 0.089 | 1.000 | ns |
| min_hb | aHUS | pregnancy_related | 16 | 158 | 3.732 | 0.000 | 0.005 | ** |
| min_hb | aHUS | malignancy | 16 | 626 | 1.097 | 0.273 | 1.000 | ns |
| min_hb | aHUS | malignant_hypertension | 16 | 21 | 1.046 | 0.296 | 1.000 | ns |
| min_hb | aHUS | STEC-HUS | 16 | 39 | 1.594 | 0.111 | 1.000 | ns |
| min_hb | aHUS | transplant | 16 | 392 | 2.803 | 0.005 | 0.106 | ns |
| min_hb | aHUS | TTP | 16 | 41 | 2.181 | 0.029 | 0.496 | ns |
| min_hb | infectious_related | pregnancy_related | 1042 | 158 | 6.444 | 0.000 | 0.000 | **** |
| min_hb | infectious_related | malignancy | 1042 | 626 | -2.992 | 0.003 | 0.064 | ns |
| min_hb | infectious_related | malignant_hypertension | 1042 | 21 | -0.371 | 0.710 | 1.000 | ns |
| min_hb | infectious_related | STEC-HUS | 1042 | 39 | 0.271 | 0.787 | 1.000 | ns |
| min_hb | infectious_related | transplant | 1042 | 392 | 4.827 | 0.000 | 0.000 | **** |
| min_hb | infectious_related | TTP | 1042 | 41 | 1.344 | 0.179 | 1.000 | ns |
| min_hb | pregnancy_related | malignancy | 158 | 626 | -7.879 | 0.000 | 0.000 | **** |
| min_hb | pregnancy_related | malignant_hypertension | 158 | 21 | -2.721 | 0.007 | 0.124 | ns |
| min_hb | pregnancy_related | STEC-HUS | 158 | 39 | -2.830 | 0.005 | 0.102 | ns |
| min_hb | pregnancy_related | transplant | 158 | 392 | -2.803 | 0.005 | 0.106 | ns |
| min_hb | pregnancy_related | TTP | 158 | 41 | -1.918 | 0.055 | 0.881 | ns |
| min_hb | malignancy | malignant_hypertension | 626 | 21 | 0.313 | 0.754 | 1.000 | ns |
| min_hb | malignancy | STEC-HUS | 626 | 39 | 1.184 | 0.236 | 1.000 | ns |
| min_hb | malignancy | transplant | 626 | 392 | 6.789 | 0.000 | 0.000 | **** |
| min_hb | malignancy | TTP | 626 | 41 | 2.266 | 0.023 | 0.422 | ns |
| min_hb | malignant_hypertension | STEC-HUS | 21 | 39 | 0.465 | 0.642 | 1.000 | ns |
| min_hb | malignant_hypertension | transplant | 21 | 392 | 1.642 | 0.101 | 1.000 | ns |
| min_hb | malignant_hypertension | TTP | 21 | 41 | 1.102 | 0.270 | 1.000 | ns |
| min_hb | STEC-HUS | transplant | 39 | 392 | 1.440 | 0.150 | 1.000 | ns |
| min_hb | STEC-HUS | TTP | 39 | 41 | 0.759 | 0.448 | 1.000 | ns |
| min_hb | transplant | TTP | 392 | 41 | -0.439 | 0.661 | 1.000 | ns |

Global Kruskal-Wallis rank sum test, p <0.001

Dunn Test adjustment to multiple comparisons with Holm (post-hoc test)

**Table 07. Multiple comparisons between delta creatinine and thrombotic microangiopathy (TMA) groups in hospitalized patients.**

| **.y.** | **group1** | **group2** | **n1** | **n2** | **statistic** | **p** | **p.adj** | **p.adj.signif** |
| --- | --- | --- | --- | --- | --- | --- | --- | --- |
| delta_creat | aHUS | infectious_related | 14 | 1033 | -1.188 | 0.235 | 1.000 | ns |
| delta_creat | aHUS | pregnancy_related | 14 | 150 | -4.818 | 0.000 | 0.000 | **** |
| delta_creat | aHUS | malignancy | 14 | 616 | -2.505 | 0.012 | 0.135 | ns |
| delta_creat | aHUS | malignant_hypertension | 14 | 17 | -0.417 | 0.677 | 1.000 | ns |
| delta_creat | aHUS | STEC-HUS | 14 | 39 | -0.496 | 0.620 | 1.000 | ns |
| delta_creat | aHUS | transplant | 14 | 372 | 1.653 | 0.098 | 0.786 | ns |
| delta_creat | aHUS | TTP | 14 | 40 | -3.729 | 0.000 | 0.003 | ** |
| delta_creat | infectious_related | pregnancy_related | 1033 | 150 | -11.750 | 0.000 | 0.000 | **** |
| delta_creat | infectious_related | malignancy | 1033 | 616 | -7.022 | 0.000 | 0.000 | **** |
| delta_creat | infectious_related | malignant_hypertension | 1033 | 17 | 0.692 | 0.489 | 1.000 | ns |
| delta_creat | infectious_related | STEC-HUS | 1033 | 39 | 1.013 | 0.311 | 1.000 | ns |
| delta_creat | infectious_related | transplant | 1033 | 372 | 12.730 | 0.000 | 0.000 | **** |
| delta_creat | infectious_related | TTP | 1033 | 40 | -5.201 | 0.000 | 0.000 | **** |
| delta_creat | pregnancy_related | malignancy | 150 | 616 | 7.350 | 0.000 | 0.000 | **** |
| delta_creat | pregnancy_related | malignant_hypertension | 150 | 17 | 4.673 | 0.000 | 0.000 | **** |
| delta_creat | pregnancy_related | STEC-HUS | 150 | 39 | 6.632 | 0.000 | 0.000 | **** |
| delta_creat | pregnancy_related | transplant | 150 | 372 | 18.573 | 0.000 | 0.000 | **** |
| delta_creat | pregnancy_related | TTP | 150 | 40 | 1.059 | 0.289 | 1.000 | ns |
| delta_creat | malignancy | malignant_hypertension | 616 | 17 | 2.142 | 0.032 | 0.290 | ns |
| delta_creat | malignancy | STEC-HUS | 616 | 39 | 3.166 | 0.002 | 0.020 | * |
| delta_creat | malignancy | transplant | 616 | 372 | 17.167 | 0.000 | 0.000 | **** |
| delta_creat | malignancy | TTP | 616 | 40 | -2.946 | 0.003 | 0.039 | * |
| delta_creat | malignant_hypertension | STEC-HUS | 17 | 39 | -0.013 | 0.989 | 1.000 | ns |
| delta_creat | malignant_hypertension | transplant | 17 | 372 | 2.422 | 0.015 | 0.155 | ns |
| delta_creat | malignant_hypertension | TTP | 17 | 40 | -3.479 | 0.001 | 0.007 | ** |
| delta_creat | STEC-HUS | transplant | 39 | 372 | 3.591 | 0.000 | 0.005 | ** |
| delta_creat | STEC-HUS | TTP | 39 | 40 | -4.459 | 0.000 | 0.000 | *** |
| delta_creat | transplant | TTP | 372 | 40 | -9.663 | 0.000 | 0.000 | **** |

Global Kruskal-Wallis rank sum test, p <0.001

Dunn Test adjustment to multiple comparisons with Holm (post-hoc test)

**Table 08. Multiple comparisons between maximum bilirubin and thrombotic microangiopathy (TMA) groups in hospitalized patients.**

| **.y.** | **group1** | **group2** | **n1** | **n2** | **statistic** | **p** | **p.adj** | **p.adj.signif** |
| --- | --- | --- | --- | --- | --- | --- | --- | --- |
| max_bt | aHUS | infectious_related | 12 | 859 | 2.256 | 0.024 | 0.457 | ns |
| max_bt | aHUS | pregnancy_related | 12 | 143 | -0.333 | 0.739 | 1.000 | ns |
| max_bt | aHUS | malignancy | 12 | 498 | 2.182 | 0.029 | 0.524 | ns |
| max_bt | aHUS | malignant_hypertension | 12 | 16 | 0.347 | 0.729 | 1.000 | ns |
| max_bt | aHUS | STEC-HUS | 12 | 24 | 1.631 | 0.103 | 1.000 | ns |
| max_bt | aHUS | transplant | 12 | 102 | -0.098 | 0.922 | 1.000 | ns |
| max_bt | aHUS | TTP | 12 | 34 | 2.294 | 0.022 | 0.436 | ns |
| max_bt | infectious_related | pregnancy_related | 859 | 143 | -8.371 | 0.000 | 0.000 | **** |
| max_bt | infectious_related | malignancy | 859 | 498 | -0.329 | 0.742 | 1.000 | ns |
| max_bt | infectious_related | malignant_hypertension | 859 | 16 | -2.075 | 0.038 | 0.608 | ns |
| max_bt | infectious_related | STEC-HUS | 859 | 24 | -0.384 | 0.701 | 1.000 | ns |
| max_bt | infectious_related | transplant | 859 | 102 | -6.550 | 0.000 | 0.000 | **** |
| max_bt | infectious_related | TTP | 859 | 34 | 0.655 | 0.513 | 1.000 | ns |
| max_bt | pregnancy_related | malignancy | 143 | 498 | 7.773 | 0.000 | 0.000 | **** |
| max_bt | pregnancy_related | malignant_hypertension | 143 | 16 | 0.882 | 0.378 | 1.000 | ns |
| max_bt | pregnancy_related | STEC-HUS | 143 | 24 | 3.067 | 0.002 | 0.048 | * |
| max_bt | pregnancy_related | transplant | 143 | 102 | 0.541 | 0.589 | 1.000 | ns |
| max_bt | pregnancy_related | TTP | 143 | 34 | 4.562 | 0.000 | 0.000 | *** |
| max_bt | malignancy | malignant_hypertension | 498 | 16 | -1.988 | 0.047 | 0.702 | ns |
| max_bt | malignancy | STEC-HUS | 498 | 24 | -0.291 | 0.771 | 1.000 | ns |
| max_bt | malignancy | transplant | 498 | 102 | -6.141 | 0.000 | 0.000 | **** |
| max_bt | malignancy | TTP | 498 | 34 | 0.750 | 0.453 | 1.000 | ns |
| max_bt | malignant_hypertension | STEC-HUS | 16 | 24 | 1.376 | 0.169 | 1.000 | ns |
| max_bt | malignant_hypertension | transplant | 16 | 102 | -0.604 | 0.546 | 1.000 | ns |
| max_bt | malignant_hypertension | TTP | 16 | 34 | 2.104 | 0.035 | 0.601 | ns |
| max_bt | STEC-HUS | transplant | 24 | 102 | -2.674 | 0.008 | 0.158 | ns |
| max_bt | STEC-HUS | TTP | 24 | 34 | 0.727 | 0.467 | 1.000 | ns |
| max_bt | transplant | TTP | 102 | 34 | 4.042 | 0.000 | 0.001 | ** |

Global Kruskal-Wallis rank sum test, p <0.001

Dunn Test adjustment to multiple comparisons with Holm (post-hoc test)

**Table 09. Multiple comparisons between maximum AST and thrombotic microangiopathy (TMA) groups in hospitalized patients.**

| **.y.** | **group1** | **group2** | **n1** | **n2** | **statistic** | **p** | **p.adj** | **p.adj.signif** |
| --- | --- | --- | --- | --- | --- | --- | --- | --- |
| max_ast | aHUS | infectious_related | 14 | 460 | 1.422 | 0.155 | 1.000 | ns |
| max_ast | aHUS | pregnancy_related | 14 | 105 | 0.447 | 0.655 | 1.000 | ns |
| max_ast | aHUS | malignancy | 14 | 341 | 1.457 | 0.145 | 1.000 | ns |
| max_ast | aHUS | malignant_hypertension | 14 | 8 | 0.978 | 0.328 | 1.000 | ns |
| max_ast | aHUS | STEC-HUS | 14 | 12 | 0.718 | 0.473 | 1.000 | ns |
| max_ast | aHUS | transplant | 14 | 296 | 0.236 | 0.813 | 1.000 | ns |
| max_ast | aHUS | TTP | 14 | 28 | 0.916 | 0.360 | 1.000 | ns |
| max_ast | infectious_related | pregnancy_related | 460 | 105 | -2.392 | 0.017 | 0.419 | ns |
| max_ast | infectious_related | malignancy | 460 | 341 | 0.160 | 0.873 | 1.000 | ns |
| max_ast | infectious_related | malignant_hypertension | 460 | 8 | 0.134 | 0.894 | 1.000 | ns |
| max_ast | infectious_related | STEC-HUS | 460 | 12 | -0.353 | 0.724 | 1.000 | ns |
| max_ast | infectious_related | transplant | 460 | 296 | -4.310 | 0.000 | 0.000 | *** |
| max_ast | infectious_related | TTP | 460 | 28 | -0.441 | 0.659 | 1.000 | ns |
| max_ast | pregnancy_related | malignancy | 105 | 341 | 2.420 | 0.016 | 0.403 | ns |
| max_ast | pregnancy_related | malignant_hypertension | 105 | 8 | 0.835 | 0.404 | 1.000 | ns |
| max_ast | pregnancy_related | STEC-HUS | 105 | 12 | 0.510 | 0.610 | 1.000 | ns |
| max_ast | pregnancy_related | transplant | 105 | 296 | -0.549 | 0.583 | 1.000 | ns |
| max_ast | pregnancy_related | TTP | 105 | 28 | 0.813 | 0.416 | 1.000 | ns |
| max_ast | malignancy | malignant_hypertension | 341 | 8 | 0.101 | 0.919 | 1.000 | ns |
| max_ast | malignancy | STEC-HUS | 341 | 12 | -0.390 | 0.696 | 1.000 | ns |
| max_ast | malignancy | transplant | 341 | 296 | -4.186 | 0.000 | 0.001 | *** |
| max_ast | malignancy | TTP | 341 | 28 | -0.495 | 0.621 | 1.000 | ns |
| max_ast | malignant_hypertension | STEC-HUS | 8 | 12 | -0.331 | 0.741 | 1.000 | ns |
| max_ast | malignant_hypertension | transplant | 8 | 296 | -1.029 | 0.303 | 1.000 | ns |
| max_ast | malignant_hypertension | TTP | 8 | 28 | -0.333 | 0.739 | 1.000 | ns |
| max_ast | STEC-HUS | transplant | 12 | 296 | -0.740 | 0.459 | 1.000 | ns |
| max_ast | STEC-HUS | TTP | 12 | 28 | 0.050 | 0.960 | 1.000 | ns |
| max_ast | transplant | TTP | 296 | 28 | 1.190 | 0.234 | 1.000 | ns |

Global Kruskal-Wallis rank sum test, p <0.001

Dunn Test adjustment to multiple comparisons with Holm (post-hoc test)

**Table 10. Most frequently primary diagnostic of infectious related thrombotic microangiopathy (TMA).**

| Diagnostic | n |
| --- | --- |
| Unspecified septicemia | 183 |
| Other specified septicemias | 162 |
| Unspecified bacterial infection | 137 |
| Coronavirus infection, unspecified site | 101 |
| Other specified bacterial diseases | 80 |
| Toxic shock syndrome | 42 |
| Unspecified bronchopneumonia | 36 |
| Unspecified pneumonia | 36 |

**Practical Examples of TMA-INSIGHT prediction**

The first example (id=1) was a 30-year-old patient with kidney disease, a minimum platelet count of 90,000/µL, LDH level of 1500, delta creatinine of 3 mg/dl, normal coagulation values, bilirubin, and AST. The mean arterial pressure was 120 mmHg, and the higher probability was attributed to aHUS.

Patient 2 was a 50-year-old with hypertension, an active infection, and altered values of AST and bilirubin. The higher probability for this patient was TMA secondary to infection.

Patient 3 was a 30-year-old with an active pregnancy, a delta creatinine of 2, and a MAP of 100 mmHg. The higher probability for this patient was TMA secondary to pregnancy.

Patient 4 was a 60-year-old with hypertension and diabetes, and an active malignancy. The higher probability for this patient was TMA secondary to malignancy.

Patient 5 was a 40-year-old with hypertensive emergency and a MAP of 120 mmHg. The higher probability for this patient was TMA secondary to malignant hypertension.

Patient 6 was a 5-year-old with no active infection (excluding gastrointestinal), a minimum platelet count of 70,000/µL, and no comorbidities. The predictions pointed to STEC-HUS.

Patient 7 was a 30-year-old with no active infection, a minimum platelet count of 90,000/µL, a delta creatinine of 4mg/dl, and active kindey transplant. The predictions pointed to TMA secondary to transplant.

Patient 8 was a 40-year-old with a delta creatinine of 1.5, a minimum platelet count of 20,000/µL, and no comorbidities. The prediction for this patient was TTP.

**Table 10. Laboratory and clinical characteristics of 8 simulated patients (id) for practical Examples of TMA-INSIGHT prediction**

| **id** | **age** | **delta_creat** | **max_LDH** | **min_plat** | **min_hb** | **max_ast** | **max_bt** | **max_ttap** | **max_tp** | **hypertension** | **hypertension_emergency** | **MAP** |
| --- | --- | --- | --- | --- | --- | --- | --- | --- | --- | --- | --- | --- |
| 1 | 30 | 3.0 | 1500 | 90 | 7 | 30 | 1.0 | 20 | 15 | 0 | 0 | 130 |
| 2 | 50 | 2.0 | 1700 | 100 | 9 | 50 | 1.5 | 35 | 20 | 1 | 0 | 80 |
| 3 | 30 | 2.0 | 1500 | 80 | 8 | 40 | 2.0 | 20 | 15 | 0 | 0 | 100 |
| 4 | 60 | 2.0 | 2000 | 90 | 7 | 50 | 2.0 | 20 | 20 | 1 | 0 | 90 |
| 5 | 40 | 3.0 | 2000 | 80 | 7 | 40 | 1.0 | 15 | 15 | 0 | 1 | 120 |
| 6 | 5 | 2.0 | 2500 | 70 | 7 | 40 | 1.0 | 15 | 15 | 0 | 0 | 100 |
| 7 | 30 | 4.0 | 2000 | 90 | 8 | 30 | 1.0 | 15 | 15 | 1 | 0 | 110 |
| 8 | 40 | 1.5 | 3000 | 20 | 7 | 25 | 1.0 | 15 | 20 | 0 | 0 | 100 |

| **id** | **diabetes** | **cardiovascular_disease** | **kidney_disease** | **liver_disease** | **transplant** | **pregnancy** | **Autoimmune_disease** | **malignancy** | **covid** | **infection** |
| --- | --- | --- | --- | --- | --- | --- | --- | --- | --- | --- |
| 1 | 0 | 0 | 1 | 0 | 0 | 0 | 0 | 0 | 0 | 0 |
| 2 | 0 | 0 | 0 | 0 | 0 | 0 | 0 | 0 | 0 | 1 |
| 3 | 0 | 0 | 0 | 0 | 0 | 1 | 0 | 0 | 0 | 0 |
| 4 | 1 | 0 | 0 | 1 | 0 | 0 | 0 | 1 | 0 | 0 |
| 5 | 0 | 0 | 0 | 0 | 0 | 0 | 0 | 0 | 0 | 0 |
| 6 | 0 | 0 | 0 | 0 | 0 | 0 | 0 | 0 | 0 | 0 |
| 7 | 1 | 0 | 1 | 0 | 1 | 0 | 0 | 0 | 0 | 0 |
| 8 | 0 | 0 | 0 | 0 | 0 | 0 | 0 | 0 | 0 | 0 |

**Table 11. TMA-INSIGHT prediction probabilities of 8 simulated patients**

| **aHUS** | **.Infectious related** | **Pregnancy related** | **.Malignancy** | **Malignant hypertension** | **STEC-HUS** | **Transplant** | **TTP** |
| --- | --- | --- | --- | --- | --- | --- | --- |
| 0.572 | 0.043 | 0.06 | 0.031 | 0.023 | 0.127 | 0.091 | 0.055 |
| 0.000 | 0.999 | 0.00 | 0.000 | 0.000 | 0.001 | 0.000 | 0.000 |
| 0.000 | 0.000 | 1.00 | 0.000 | 0.000 | 0.000 | 0.000 | 0.000 |
| 0.000 | 0.000 | 0.00 | 1.000 | 0.000 | 0.000 | 0.000 | 0.000 |
| 0.000 | 0.000 | 0.00 | 0.000 | 1.000 | 0.000 | 0.000 | 0.000 |
| 0.001 | 0.000 | 0.00 | 0.000 | 0.000 | 0.999 | 0.000 | 0.000 |
| 0.067 | 0.000 | 0.00 | 0.000 | 0.000 | 0.008 | 0.924 | 0.000 |
| 0.000 | 0.000 | 0.00 | 0.000 | 0.000 | 0.000 | 0.000 | 1.000 |
